# Single Particle Tracking Reveals that EGFR Signaling Activity Is Amplified in Clathrin-Coated Pits

**DOI:** 10.1371/journal.pone.0143162

**Published:** 2015-11-17

**Authors:** Jenny Ibach, Yvonne Radon, Márton Gelléri, Michael H. Sonntag, Luc Brunsveld, Philippe I. H. Bastiaens, Peter J. Verveer

**Affiliations:** 1 Department of Systemic Cell Biology, Max Planck Institute of Molecular Physiology, Dortmund, Germany; 2 Laboratory of Chemical Biology, Department of Biomedical Engineering, and Institute of Complex Molecular Systems, Eindhoven University of Technology, Eindhoven, The Netherlands; Thomas Jefferson University, UNITED STATES

## Abstract

Signaling from the epidermal growth factor receptor (EGFR) via phosphorylation on its C-terminal tyrosine residues requires self-association, which depends on the diffusional properties of the receptor and its density in the plasma membrane. Dimerization is a key event for EGFR activation, but the role of higher order clustering is unknown. We employed single particle tracking to relate the mobility and aggregation of EGFR to its signaling activity. EGFR mobility alternates between short-lived free, confined and immobile states. In the immobile state, EGFR tends to aggregate in clathrin-coated pits, which is further enhanced in a phosphorylation-dependent manner and does not require ligand binding. EGFR phosphorylation is further amplified by cross-phosphorylation in clathrin-coated pits. Because phosphorylated receptors can escape from the pits, local gradients of signaling active EGFR are formed. These results show that amplification of EGFR phosphorylation by receptor clustering in clathrin-coated pits supports signal activation at the plasma membrane.

## Introduction

The epidermal growth factor receptor (EGFR, or ErbB1) translates extracellular cues into an intracellular response that triggers a specific physiological output such as proliferation or migration, depending on the cellular context [1,2]. Aberrant activation of EGFR is implicated in tumor development [3] and its activation mechanisms have been a topic of intense study [4,5]. EGFR responds to the binding of ligand by forming dimers, which activates its kinase domains by an allosteric mechanism [6]. Dimerization is a key initial step, which was demonstrated initially by biochemical experiments [7], and later confirmed in living cells by single molecule imaging [8]. Structural studies showed that the transmembrane and juxtamembrane segments favor dimerization [9–11], which is sterically constrained by the extracellular domain [4]. Ligand binding lifts that constraint by changing the configuration of the extracellular domain from a tethered to an extended geometry [4,12,13]. In the ligand-bound dimer, the intracellular kinase domains form an asymmetric dimer, with the ‘activator’ kinase functioning as an allosteric activator of the ‘receiver’ kinase [6]. This leads to phosphorylation of multiple residues in the cytoplasmic tail of the activator receptor, which serve as docking sites for effectors. These structural studies show a central role for receptor dimerization in the activation of the kinase domains. However, it is not clear if the dimer is also the fundamental unit in terms of the signaling activity of the phosphorylated receptor. For instance, because EGFR dimerization is transient [14–16] also the phosphorylated monomer could contribute to the signal. Higher order EGFR clusters have also been reported [17–23] but their role in the signaling activity of EGFR is largely unexplored. Higher order clusters have not been studied on a structural level, and it is not clear if EGFR clustering is an intrinsic structural property of the ligand-bound receptor or if it depends on interactions with extrinsic factors other than ligand.

EGFR can be phosphorylated in the absence of ligand in spite of the structural constraints that prevent activation of the kinase domains [14,24]. This is possibly supported by an autocatalytic mechanism where phosphorylation of Tyr845 suppresses the intrinsic disorder of the αC helix in the kinase domain, which leads to a higher dimerization efficiency [25]. To prevent spurious signaling activity of EGFR, phosphorylation of the receptor is curbed extrinsically by protein tyrosine phosphatases [14,26–29]. However, this raises the question if activation of the intrinsic kinase activity of the receptor by ligand binding is sufficient to generate a stable level of receptor phosphorylation at the plasma membrane, since the activity of protein tyrosine counteracted phosphatases is two orders of magnitude higher [30,31]. Additional mechanisms that promote EGFR phosphorylation, for instance by spatially tuning the activity of protein tyrosine phosphatases [32], could compensate for this.

Here we investigate if the distribution of the receptor in the plasma membrane is modulated upon ligand binding in order to support phosphorylation of EGFR in a background of phosphatase activity. In particular, an increase in the local concentration of the receptor by clustering could support EGFR activation at low expression levels by increasing the local kinase density. We employed single particle tracking to study the mobility and aggregation of EGFR, and correlated this with its signaling activity by simultaneously detecting the binding of a phosphotyrosine-binding (PTB) domain. We find that the mobility of EGFR alternates between free, confined and immobile states, on a time scale of seconds. After phosphorylation, EGFR tends to aggregate in the immobile state in clathrin-coated pits, where its autophosphorylation is amplified. The clustering of EGFR depends on its phosphorylation and does not require ligand binding. The receptors are able to escape the clathrin-coated pits, which establishes local gradients of mobile phosphorylated EGFR. Interfering with receptor recruitment to clathrin-coated pits substantially delays signal activation, which shows that amplification of receptor phosphorylation in clathrin-coated pits supports EGFR signaling at the plasma membrane.

## Materials and Methods

### Reagents

Human EGF was purchased from Peprotech (Hamburg, Germany) and shock frozen at 100 μg/ml in PBS + 0.1% BSA. Benzylguanine-Cy3 (BG-Cy3) was synthesized by chemically coupling with BG-NH_2_ (NEB, Frankfurt am Main, Germany) and mono-reactive Cy3 NHS ester (Amersham, GE Healthcare, Freiburg, Germany). A 1 mM stock solution in DMSO was kept at -20°C. Benzylguanine-Alexa488 (BG-Alexa488) was purchased (NEB, Frankfurt am Main, Germany). Site-specific *C*-terminal labeling of hEGF-Cys was carried out as described previously [33]. Briefly, the unprotected *C*-terminal cysteine in hEGF-Cys was labeled with a 5 times excess of Alexa488-maleimide (Invitrogen, Life Technologies GmbH, Darmstadt, Germany) in aqueous solution (PBS, pH7.4) under argon for 2 hours at 4°C in the dark.

### Constructs

To clone SNAP-EGFR, the EGFP in EGFP-EGFR was replaced by a SNAP-tag. The sequence for SNAP was amplified by PCR from pSNAP-tag(m) (NEB, Frankfurt am Main, Germany) with the primers SNAP_AgeI_F (GCG ACC GGT AAT GGA CAA AGA CTG CGA AAT G) and SNAP_XhoI_R (CTC CTC GAG CAC CCA GCC CAG GCA), digested with AgeI (NEB, Frankfurt am Main, Germany) and XhoI (NEB, Frankfurt am Main, Germany) and cloned into the AgeI/XhoI restriction sites of the digested EGFP-EGFR plasmid. The point mutation Y1045F was introduced into SNAP-EGFR with the primers Y1045F_F (AGC TTC TTG CAG CGA TTC AGC TCA GAC CCC ACA GGC) and Y1045F_R (TCG AAG AAC GTC GCT AAG TCG AGT CTG GGG TGT CCG). EGFP-PTB and EGFP-clathrin were described previously [34].

### Cell culture and sample preparation

MCF-7 cells (obtained from ATCC) were grown in DMEM (PAN Biotech, Aidenbach, Germany) supplemented with 10% (v/v) Fetal Calf Serum (FCS, Life Technologies GmbH, Darmstadt, Germany), 1% L-glutamine (PAN Biotech, Aidenbach, Germany) and 1% non-essential amino acids (NEAA, PAN Biotech, Aidenbach, Germany) at 37°C and 5% CO_2_. For microscopy experiments cells were seeded in eight-well Lab-Tek chambers (Nunc, Thermo Fisher Scientific, Waltham, USA) at a density of 3×10^4^ cells per well 24 hours before transfection. Cells were transfected with Effectene (QIAGEN, Hilden, Germany) transfection reagent according to the protocol of the manufacturer with a ratio of 1:10 (DNA:effectene) and a ratio of EGFR plasmid to EGFP-PTB or EGFP-clathrin plasmid of 2:1. 6-8 hours after transfection, cells were serum starved (0% FCS) for 16–20 hours. SNAP-EGFR expressing cells were labeled with 500 nM BG-Cy3 for 5 minutes in imaging medium (DMEM without phenol red, with stable glutamine and 25 mM Hepes, PAN Biotech, Aidenbach, Germany) + 0.5% BSA at 37°C, then washed three times with imaging medium + 0.5% BSA. For dual-color tracking of the receptor, cells were labeled with 250 nM of BG-Cy3 and 250 nM of BG-Alexa488. Directly before the measurements the medium was removed, cells were washed twice with DPBS+ (DPBS with magnesium and calcium, PAN Biotech, Aidenbach, Germany) and imaged in DPBS+. To stimulate with 16 nM EGF (100 ng/ml) or 16 nM EGF-Alexa488 (113 ng/ml) 10 μl of a 20× EGF solution was added to cells in 190 μl DPBS+. For pervanadate stimulation, 8 μl of a freshly prepared 25 mM pervanadate solution (25 mM sodium orthovanadate (Sigma-Aldrich, Taufkirchen, Germany) mixed with 12.5 mM H_2_O_2_ (Sigma-Aldrich, Taufkirchen, Germany) in ddH_2_O) was added to cells in 192 μl DPBS+. For experiments with dynasore (Calbiochem, Merck KGaA, Darmstadt, Germany), cells were washed twice with DPBS+, 190 μl of an 80 μM dynasore solution in DPBS+ was added to the cells, and incubated for 30 minutes prior to stimulation and imaging.

### Confocal microscopy

Confocal microscopy was done with a Leica SP5 inverted confocal microscope (Leica Microsystems GmbH, Wetzlar, Germany) equipped with a 63x oil objective (HCX PL APO lambda blue 1.4NA) and an incubation chamber at 37°C and 5% CO_2_. Dual-color data were acquired by sequential imaging: Channel 1: (EGFP/Alexa488) excitation with 476 nm, emission 500–550 nm. Channel 2: (Cy3) excitation with 561 nm, emission 575-625 nm.

### Total internal reflection microscopy

Single molecule tracking data were acquired with an Olympus IX81 inverted microscope equipped with a total internal reflection fluorescence (TIRF) illuminator (Olympus GmbH, Hamburg, Germany). Laser illumination was provided by an argon-ion laser (Innova Sabre DBW 20, Coherent Inc., Dieburg, Germany) emitting at 476 nm, and a krypton-ion laser (Innova Sabre DBW, Coherent Inc., Dieburg, Germany) emitting at 568 nm, each coupled into the TIRF illuminator via a single mode optical fiber. Both lasers were set to emit at an intensity of 100 mW. We estimated that due to losses in the optical setup about 12% of the illumination power reached the sample. Fluorescence was collected with a PLAPON60xO/TIRFM-SP Objective (NA = 1.45). The emission light was spectrally separated by an Optosplit II image splitter (Cairn Research, Faversham, UK) and recorded with an Andor iXon+ DU-897 back-illuminated electron-multiplying CCD camera (BFIOptilas, Andor, Belfast, Northern Ireland). The Optosplit II was equipped with a 570LP dichroic mirror (DM570, Olympus GmbH, Hamburg, Germany), with two emission filters: 500-550 (FF01-525/50, AHF, Tübingen, Germany) and 575-625 (BP575-625, Olympus GmbH, Hamburg, Germany), and with optics to magnify the image to an effective pixel size of 107 nm. For dual-color imaging the excitation was alternated between the 476 nm and 568 nm sources, using custom build electronics to trigger the shutters in synchronization with the EM-CCD camera. The sample was maintained at a constant temperature of 37°C using a Bioptechs objective heater (Chromaphor, Oberhausen, Germany). For single particle tracking, 300 images were acquired, with an exposure time of 31.6 ms, at a rate of 30 frames per second (fps) for an effective frame rate of 15 fps per channel. In a single measurement on the average 2×10^3^ tracks were collected.

### Analysis of clathrin fluorescence distribution

The distribution of EGFP-clathrin fluorescence was quantified using the Fiji image-processing package [35]. Background fluorescence was subtracted, the images were segmented, and the resulting spots were counted and quantified by measuring their average size and fluorescence intensity per cell. The spot density was calculated by dividing the spot count by the cell area.

### Single molecule tracking data analysis and classification

A custom MATLAB (Mathworks, Natick MA) script was used to separate and align the channels of each frame of a sequence. The shifts between channels were derived from images of TetraSpeck fluorescent microsphere standards (Thermo Fisher Scientific, Waltham, MA), which can be imaged with high signal-to-noise ratios. The shifts were determined using the MATLAB ‘imregtform’ command, which was able to register the channels with a precision of about 5 nanometers. Cells were selected manually using the Fiji image-processing package [35]. Single particle tracks were obtained using the u-track package [36], and tracks within the selected areas were further analyzed with the vbSPT package [37] to classify each localization according to a 3-state model. Simulations based on a 3-state model, consisting of free, confined and immobile states, confirmed that the vbSPT was able to correctly fit such models. Only tracks with a length of at least 10 localizations were selected for further analysis with MATLAB. Mean-squared-displacement analysis was done as in [38], after segmenting the tracks by state. Diffusion coefficients were derived from linear fits to the first 5 points of the MSD curves. The diffusion coefficients calculated by vbSPT do not incorporate corrections for the localization error and were not used. The duration of each state was derived from the mean of the number of particle localizations in each track segment.

### Single molecule colocalization analysis

A particle detected in the second channel was determined to colocalize with a particle acquired in the first channel, if it was found within the area it could explore during acquisition of a single frame. This is equivalent to finding the distance one particle may travel within one frame acquisition. The mean-squared-distance over one acquisition frame is given by [38]:

$$MSD=\left(4\sigma^{2}-\frac{4}{3}D\Delta t\right)+4D\Delta t,$$ (1)

where *σ* is the standard deviation of the localization error, *D* is the diffusion coefficient, and Δ*t* is the time lag between two frames. The localization error *σ* can vary per particle, but since this information is not available, we use the estimated average localization error of 20 nm for our system [39]. We approximated the probability of finding the particle at a given distance by a normal distribution with a variance equal to the MSD. A 95% confidence level was then used to find a threshold to determine if two particles in the different channels were colocalizing. Using the diffusion coefficients in unstimulated cells, we found for the thresholds in each state: *T*_*free*_ = 248 nm, *T*_*confined*_ = 172 nm, and *T*_*immobile*_ = 122 nm. Colocalization events were retained if particles where colocalizing for at least 5 consecutive frames, indicating that the particles where moving together. The probabilities for detecting a colocalization event were calculated by normalizing the number of colocalization events by the number of particles in the given state. In the case of dual-color tracking of SNAP-EGFR labeled with BG-Cy3 and BG-Alexa488, we applied the tracking and vbSPT analysis to the Cy3 data, and colocalized the Alexa488-labeled particles, as above. The reason for selecting Cy3 for tracking is that it is more photo-stable than Alexa488. The average lifetime of the fluorophores was derived from plots of the total intensities of the frames of a data sequence, which closely followed an exponential distribution. The average lifetime of Cy3 under our imaging conditions was 30 seconds or more, considerably larger than our acquisition time (10 s), allowing us to neglect photobleaching. However, Alexa488 had an average lifetime (~13 s) on the same order as our acquisition time, likely leading to artifacts in the tracking and vbSPT analysis. PTB was fused to EGFP, which is considerably less photo-stable than Cy3. However, because photo-bleaching of EGFP-PTB is independent of the mobility state of the receptor, this affects the colocalization in each state equally. Therefore, we were able to use colocalization with EGFP-PTB as a relative measure of EGFR phosphorylation in the different mobility states. Other endogenous proteins that contain the PTB domain, such as Shc may compete with EGFP-PTB for binding to the receptor. This also will occur equally in each state and does not affect the quantification of the relative binding probabilities.

### Spatial distribution analysis

To quantify the spatial distribution of confined and free particles in the vicinity of immobile particles, we adapted Ripley’s *K* function [40]. The standard *K* function is defined as

$$K\left(t\right)=\frac{\lambda^{-1}}{N}\sum_{j}\sum_{j\neq i}I\left(d_{ij}<t\right),$$ (2)

where *λ* is the density of the points. The sums are over all *N* points, *d*_*ij*_ is the Euclidean distance between the *i* ^th^ and the *j* ^th^ point, and *I*(*x*) is the indicator function which is equal to one if its operand *x* is true, or zero otherwise. Upon clustering, a plot of the *K* function will show a steep increase compared to a plot for randomly distributed points. We modified this equation to determine if the local density of free or confined particles is higher around immobile points:

$$K_{free}\left(t\right)=\frac{\lambda_{free}^{-1}}{N_{immobile}}\sum_{i=1}^{N_{immobile}}\sum_{j=1}^{N_{free}}I\left(d_{ij}<t\right),$$ (3)

$$K_{confined}\left(t\right)=\frac{\lambda_{confined}^{-1}}{N_{immobile}}\sum_{i=1}^{N_{immobile}}\sum_{j=1}^{N_{confined}}I\left(d_{ij}<t\right),$$ (4)

where *λ*_*free*_ and *λ*_*confined*_ are the densities of the free and confined points, *N*_*immobile*_ is the number of immobile points and *N*_*free*_ and *N*_*confined*_ are the number of free and confined points. Plots of these functions will have a steep increase compared to a plot for random points, if there is a tendency of the free or confined points to cluster around the immobile points. We calculated *K*_*free*_ or *K*_*confined*_ for each data set and calculated the mean and standard error of the resulting curves. To calculate the curves, the particle positions from all frames in the acquisition were combined. To evaluate how much the results deviate from random distributions, we randomly redistributed all particles in each data set and calculated the mean and standard errors of *K*_*free*_ or *K*_*confined*_.

### Clathrin colocalization analysis

To quantify colocalization between a set of SNAP-EGFR particles and the fluorescence image of EGFP-clathrin we adapted the equation of Manders [41], which colocalizes the intensities of a red channel *R* with a green channel *G*:

$$M_{1}=\frac{\sum_{i}R_{i,coloc}}{\sum_{i}R_{i}},$$ (5)

where *R*_*i*,*coloc*_ = *G*_*i*_ if *G*_*i*_ > 0. *M*_*1*_ measures the amount of fluorescence of the colocalizing objects relative to its total fluorescence. In our case we count the number of particles where the clathrin signal *C* is greater than zero. We set *R*_*i*_ equal to the number of particles in pixel *i* to obtain:

$$P=\frac{\sum_{i}R_{i,coloc}}{\sum_{i}R_{i}}=\frac{N_{coloc}}{N},$$ (6)

where *N*_*coloc*_ is the number of particles located in the pixels *i* where *C*_*i*_ > 0, and *N* is the total number of particles. However, this measure is highly sensitive to background signals. In addition, the clathrin signal is blurred by diffraction, and a large number of particles will erroneously be assigned to the colocalizing fraction. Therefore we replaced the requirement *C*_*i*_ > 0 by a weighting factor that assigns a higher probability of colocalization to high clathrin intensities:

$$P=\frac{\sum_{i\in S}R_{i}C_{i}}{N},$$ (7)

where *S* is the set of pixels that contain on or more localizations. This measure depends on the absolute intensities *C*_*i*_. For randomly distributed particles, we could interpret this equation as randomly drawing *N* pixels, adding their intensities and dividing by *N*. Thus, for sufficiently large values of *N*, *P* = <*C*>, the average intensity of the image *C*. Therefore we redefined the colocalization index, such that for randomly distributed particles *P* = 0:

$$P=\frac{\sum_{i\in S}R_{i}C_{i}}{N\langle C\rangle }-1.$$ (8)

## Results

### Single molecule imaging of EGFR mobility and signaling activity

**Fig 1:**
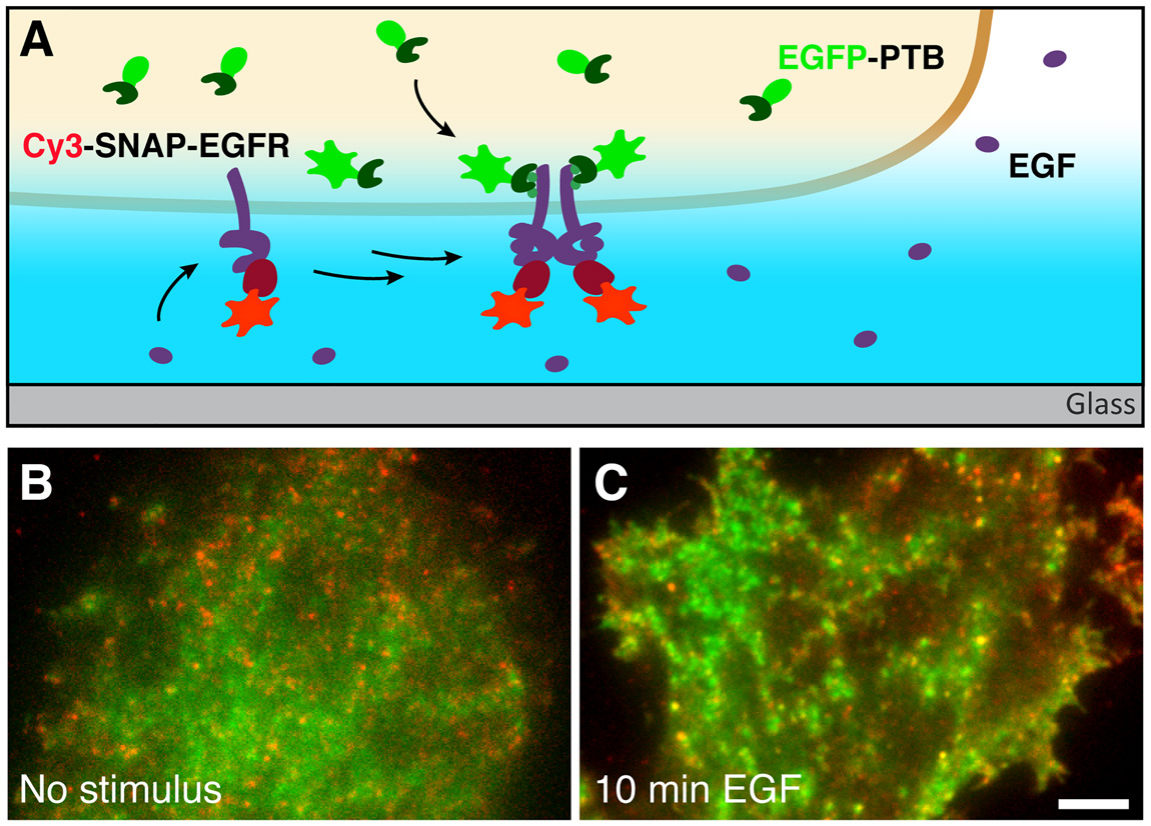
Single molecule tracking of EGFR activity. (*A*) Detection scheme for tracking EGFR activity at a single molecule level with TIRF. Single Cy3-SNAP-EGFR particles can be observed in the TIRF illumination field. Cytosolic EGFP-PTB contributes to a blurred background but does not interfere with the detection of EGFP-PTB particles that bind to the basal plasma membrane. (*B*) A single frame of a dual-color time-series acquired in an unstimulated MCF-7 cell expressing EGFP-PTB (*green*) and SNAP-EGFR labeled with Cy3 (*red*). (*C*) A single frame of a dual-color time-series acquired after 10 minutes of stimulation with 16 nM EGF in a MCF-7 cell expressing EGFP-PTB (*green*) and SNAP-EGFR labeled with Cy3 (*red*). Scale bar is 5 μm.

To investigate the mobility and the signaling activity of EGFR in living cells we employed dual-color single particle tracking. We fused a SNAP-tag [42] to the extracellular domain of EGFR (SNAP-EGFR) and ectopically expressed the receptor in MCF-7 cells. Before imaging, SNAP-tags that were exposed on the surface of the cell were labeled with the organic fluorophore Cy3 (Cy3-SNAP-EGFR). Because autophosphorylation is an early step in EGFR signaling, the signaling activity of the receptor can be probed by detecting the binding of effectors to phosphorylated tyrosine residues in its C-terminal tail. Therefore, we co-expressed the PTB domain of Shc, which binds to three phosphorylated tyrosine residues (Tyr1086, Tyr1114 and Tyr1148) in the cytoplasmic tail of EGFR [43,44], and was tagged with enhanced green fluorescent protein (EGFP-PTB). Because it only contains the phosphotyrosine binding domain of Shc, EGFP-PTB can be used as a probe for EGFR signaling activity with minimal perturbation of the downstream signal. Confocal microscopy confirmed that EGFP-PTB was rapidly recruited to the plasma membrane in ligand-stimulated cells expressing SNAP-EGFR (S1 Fig). We used dual-color total internal reflection fluorescence (TIRF) microscopy to specifically image single Cy3-SNAP-EGFR and EGFP-PTB particles at the basal plasma membrane of the cell, before and after stimulation with EGF (Fig 1A). The fluorescent particles observed in the TIRF field represent either single molecules, or consist of complexes of multiple molecules that diffuse together. To establish how EGF diffuses under the basal plasma membrane of a cell, we imaged Cy3-SNAP-EGFR together with EGF-Alexa488 (S2A Fig). In the first minute after stimulation, the ligand accumulated at the periphery of the cell, but after two minutes it reached most of the basal plasma membrane, as confirmed by colocalization analysis (S2B Fig). This corroborates an earlier report of slow EGF diffusion under the basal plasma membrane [13], and shows that after 2 minutes most receptors are exposed to the ligand. In cells with low expression levels (S3A Fig), the density of receptors in the plasma membrane was sufficiently low (~0.3 particles/μm^2^, S3B Fig) to observe the movements of individual EGFR particles. In unstimulated cells, the receptor showed a broad range of diffusive behaviors, ranging from fast movement to confinement and immobilization, and the EGFP-PTB signal mostly originated from the cytosol (S1 Movie and Fig 1B). Some plasma-membrane structures were visible that did not correlate to the distribution of Cy3-SNAP-EGFR, likely due to the binding of EGFP-PTB to phosphorylated proteins other than the receptor. After 10 minutes of stimulation with EGF a slight decrease in receptor mobility was visible and, strikingly, EGFP-PTB colocalized clearly with bright immobile spots of Cy3-SNAP-EGFR (S1 Movie and Fig 1C). EGFP-PTB was also recruited to the plasma membrane at sites where no Cy3-SNAP-EGFR was visible. There it likely binds endogenous EGFR, unlabeled SNAP-EGFR, photo-bleached Cy3-SNAP-EGFR, or other activated receptors that bind the PTB domain of Shc. Nevertheless, the striking colocalization of EGFP-PTB with bright immobile spots of Cy3-SNAP-EGFR demonstrates signaling activity from aggregated receptors.

### Ligand binding shifts free EGFR towards the immobile population

**Fig 2:**
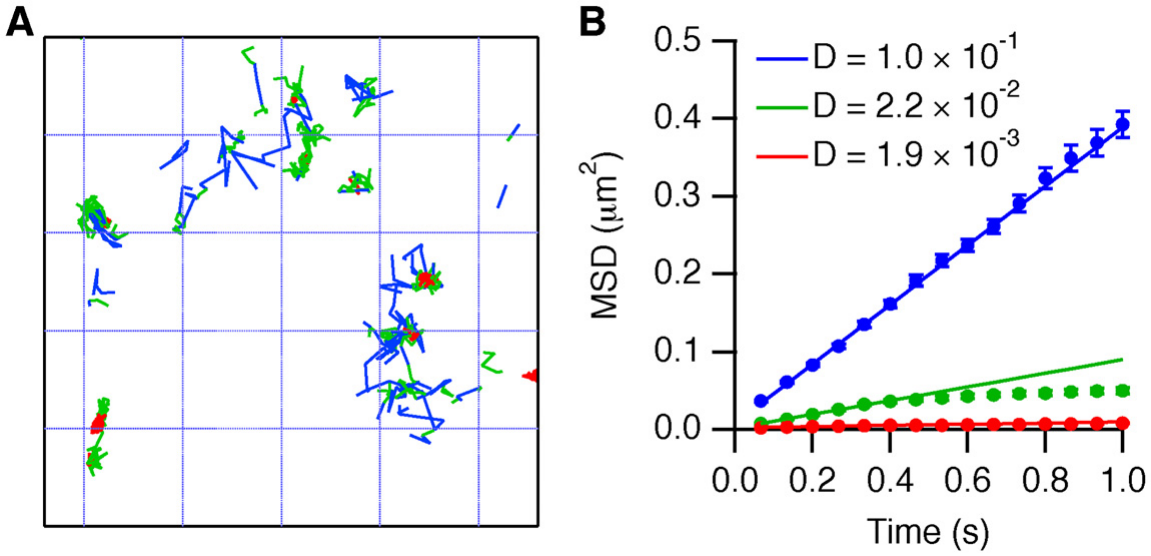
Characterization of the mobility states of EGFR. (*A*) Classified tracks of Cy3-SNAP-EGFR. The track segments are color-coded according to their mobility state (*blue*: free; *green*: confined; *red*: immobile). Shown is a 5×5 μm region of interest in an MCF-7 cell after 10 minutes of stimulation with 16 nM EGF (see also S4 Fig). (*B*) MSD analysis by state, after 10 minutes of stimulation with 16 nM EGF. The linear curves (*blue symbols*) are typical for Brownian motion, indicating free diffusion on this time-scale. The *green symbols* show a flattening of the curve due to confinement of the particles within a limited area. The *red symbols* show only minor displacement on this time-scale, indicating that the particles are immobile. *Lines*: linear fits to the beginning of each curve. Legend: fitted diffusion coefficients (*D*, μm^2^s^-1^). Error bars denote SEM.

We proceeded to analyze the mobility and activation of EGFR, up to the time point of 10 minutes where clear aggregation was observed (Fig 1C). We used the u-track package [36] to track the movement of Cy3-SNAP-EGFR (S2 Movie) and applied variational Bayes single particle tracking (vbSPT) analysis [37]. The latter models particle movements as memory-less jumps between different mobility states, where the number of states is determined by a maximum-evidence criterion that balances the goodness of fit with the complexity of the model [37]. For each cell analyzed, vbSPT yielded at least three states, and therefore we first investigated the properties of a three-state model. We divided the tracks in segments that were classified by mobility state (Fig 2A, S4 Fig) and calculated mean squared displacement (MSD) curves for each state (Fig 2B, S5 Fig), which revealed “free”, “confined” and “immobile” modes of mobility. For many cells vbSPT could also fit four or five states to the data, but MSD analysis showed that this did not introduce different modes of mobility and often led to degenerate mobility states (S6 Fig). Therefore, only three-state models are consistently supported by all data.

**Fig 3:**
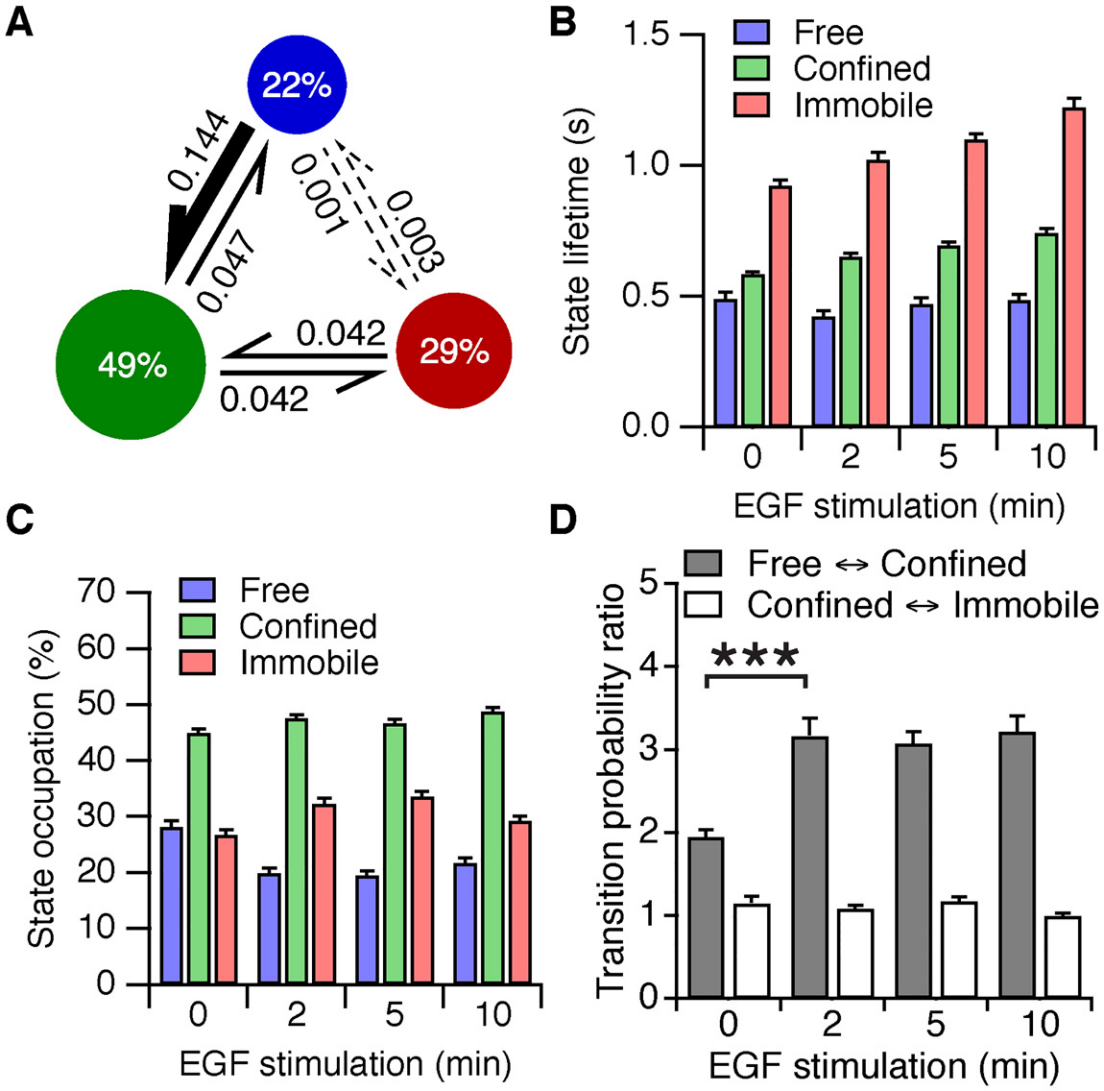
vbSPT analysis of Cy3-SNAP-EGFR tracks. (*A*) Results of the vbSPT algorithm after 10 minutes of stimulation with 16 nM EGF. *Circles*: percentages of particles in the state (state occupations). *Arrows*: probabilities to switch to another state between frames (transition probabilities). *Dashed arrows* indicate transition probabilities < 0.01. (*B*) Lifetime of the mobility states, as a function of EGF stimulation, calculated from the classified track segments. (*C*) State occupations as a function of EGF stimulation. (*D*) The ratio of the forward to the backward probabilities, for the transitions between the free and the confined states, and for the transitions between the confined and the immobile states. ***P < 0.001, *t* test. *n* = 43 cells per time point. Error bars denote SEM.

The results of the EGFR diffusion analysis are represented as state diagrams (Fig 3A, S7 Fig) displaying the relative number of particles in each state (state occupations) and the probabilities of converting between states (transition probabilities). It is important to keep in mind that these diagrams do not represent a closed system at equilibrium: the number of particles can change by fluorophore bleaching, by receptor internalization and recycling, or by assembly or disassembly of aggregates. As a result, the state occupations and the transition probabilities are not trivially related and are not easily interpreted. It should also be noted that the results of vbSPT analysis in a two-dimensional reaction-diffusion system should be interpreted with care [3]). For these reasons we mainly used vbSPT to classify the detected particles in the free, confined or immobile states before further analysis. Nevertheless, a number of important conclusions can be drawn from these state diagrams: The transitions between states were reversible, and this remained the case after stimulation with EGF, indicating that the receptor was continuously switching between different mobility states. To quantify this, we used the classified track segments to calculate the lifetimes of the three mobility states (Fig 3B). The average lifetime of each state was remarkably short, approximately half a second in the free state, with a notably longer lifetime of about one second for the immobile state, indicating that after stimulation EGFR still alternates between short-lived mobility states. The lifetime of the confined and immobile states increased by ~30% over 10 minutes of EGF stimulation, whereas the lifetime of the free state remained stable. Since the transition probabilities did not change appreciably after 2 minutes, the increase in state lifetimes of the confined and immobile states is likely the result of aggregation of the receptor.

The transition probabilities between the free and immobile states were low compared to the other transition probabilities (Fig 3A, S7 Fig). Therefore, a free particle must pass through an intermediate confined state to reach the immobile state. The most significant changes occurred after two minutes of ligand stimulation, corresponding with the time-scale of EGF binding (see S2 Fig). The occupations of the free and immobile states decreased and increased respectively, while the occupation of the confined state was not affected (Fig 3C). The ratio of the transition probabilities between the free and confined states increased (Fig 3D), showing that ligand binding drives free particles towards the confined state. The transition probabilities between the confined and immobile states were not affected by EGF stimulation, indicating that ligand binding does not directly affect the immobilization of the receptor. We conclude that ligand binding drives activated receptors into a longer-lived confined state, where their movements are likely restricted by the cortical cytoskeleton [16]. The confined active receptors are subsequently captured within immobile structures, such as clathrin-coated pits [22,45]. Because of the strong colocalization of EGFP-PTB with bright immobile Cy3-SNAP-EGFR particles (S1 Movie and Fig 1C), we next investigated the aggregation and signaling activity of EGFR in the immobile state.

### EGFR forms higher-order clusters in the immobile state

**Fig 4:**
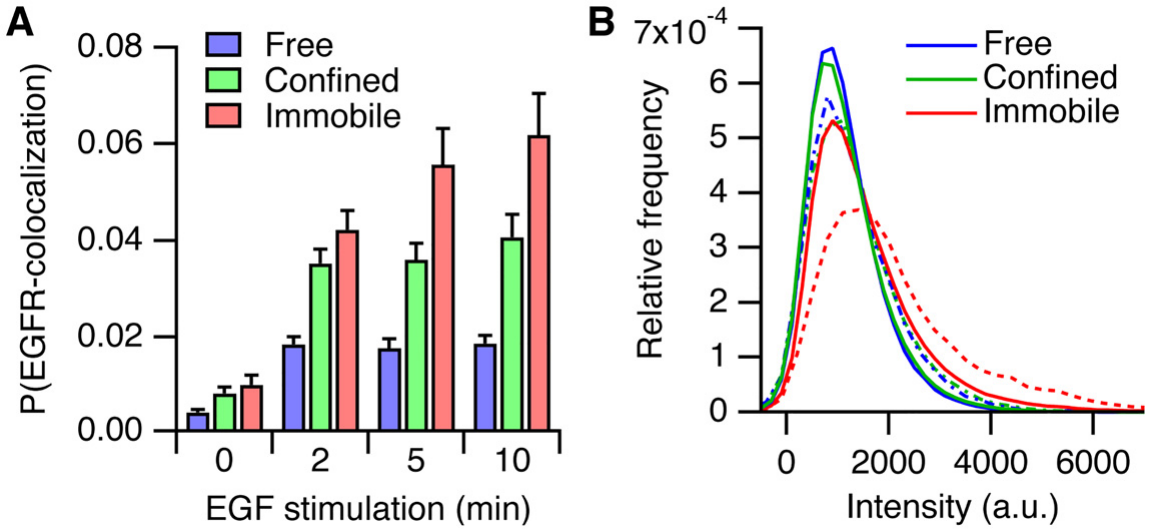
Quantification of EGFR aggregation. (*A*) Probability of colocalization between Cy3-SNAP-EGFR and Alexa488-SNAP-EGFR. (*B*) Normalized intensity histograms of the Cy3-SNAP-EGFR intensity, for all particles (*continuous lines*) and for the particles that colocalized with Alexa488-SNAP-EGFR (*dashed lines*) after 10 minutes of EGF stimulation. *n* = 43 cells per time point. Error bars denote SEM.

The aggregation of EGFR in the different mobility states was investigated by tracking SNAP-EGFR labeled with equal amounts of Cy3 and Alexa488 (S3 Movie). Aggregation of EGFR was inferred from colocalization between particles in the two channels. Particles in close proximity were considered to be colocalizing only when they moved together in the plasma membrane (see Materials and Methods), which indicates either a physical interaction or aggregation in the same compartment. We estimated the probability of colocalization by normalizing the number of colocalization events between Cy3-SNAP-EGFR and Alexa488-SNAP-EGFR particles with the number of detected Cy3-SNAP-EGFR particles in each state (Fig 4A). We detected only low levels of aggregation in unstimulated cells but given the allosteric nature of EGFR dimerization this was expected at low expression levels and corroborates results from others [22]. After stimulation with EGF the receptor aggregated, mostly in the confined and immobile states. The probability of receptor colocalization was higher in the immobile state compared to the free and confined states, either owing to a larger fraction of dimers in the immobile state, or due to the presence of higher order clusters. To differentiate between these two possibilities we inspected the fluorescence intensity histograms of Cy3-SNAP-EGFR in the different states (Fig 4B). The histograms of immobile particles were shifted and skewed towards higher intensities compared to the histograms of free and confined particles, confirming a higher degree of aggregation (Fig 4B, *solid lines*; sample mean values: μ_free_ = 1137, μ_*confined*_ = 1162, μ_*immobile*_ = 1530; sample skewness values: *s*_*free*_ = 1.4, *s*_*confined*_ = 1.4, *s*_*immobile*_ = 2.1). We also plotted intensity histograms after selecting for Cy3-SNAP-EGFR particles that colocalized with Alexa488-SNAP-EGFR, which represent the fluorescence intensities from dimers or higher order clusters (Fig 4B, *dashed lines*). The intensity histogram of the immobile colocalized particles was clearly shifted and skewed compared to the intensity histograms of the colocalized free and confined particles, indicating the presence of clusters of immobile particles consisting of three or more receptors (sample mean values: μ_free_ = 1423, μ_*confined*_ = 1474, μ_*immobile*_ = 2282; sample skewness values: *s*_*free*_ = 1.2, *s*_*confined*_ = 1.3, *s*_*immobile*_ = 1.6). Activation of EGFR by inhibition of protein tyrosine phosphatases with pervanadate was slower, requiring us to measure time-points up to 30 minutes, but showed similar results, showing that aggregation of the receptor did not require ligand binding (S8 Fig).

### EGFR phosphorylation is amplified in the immobile state

**Fig 5:**
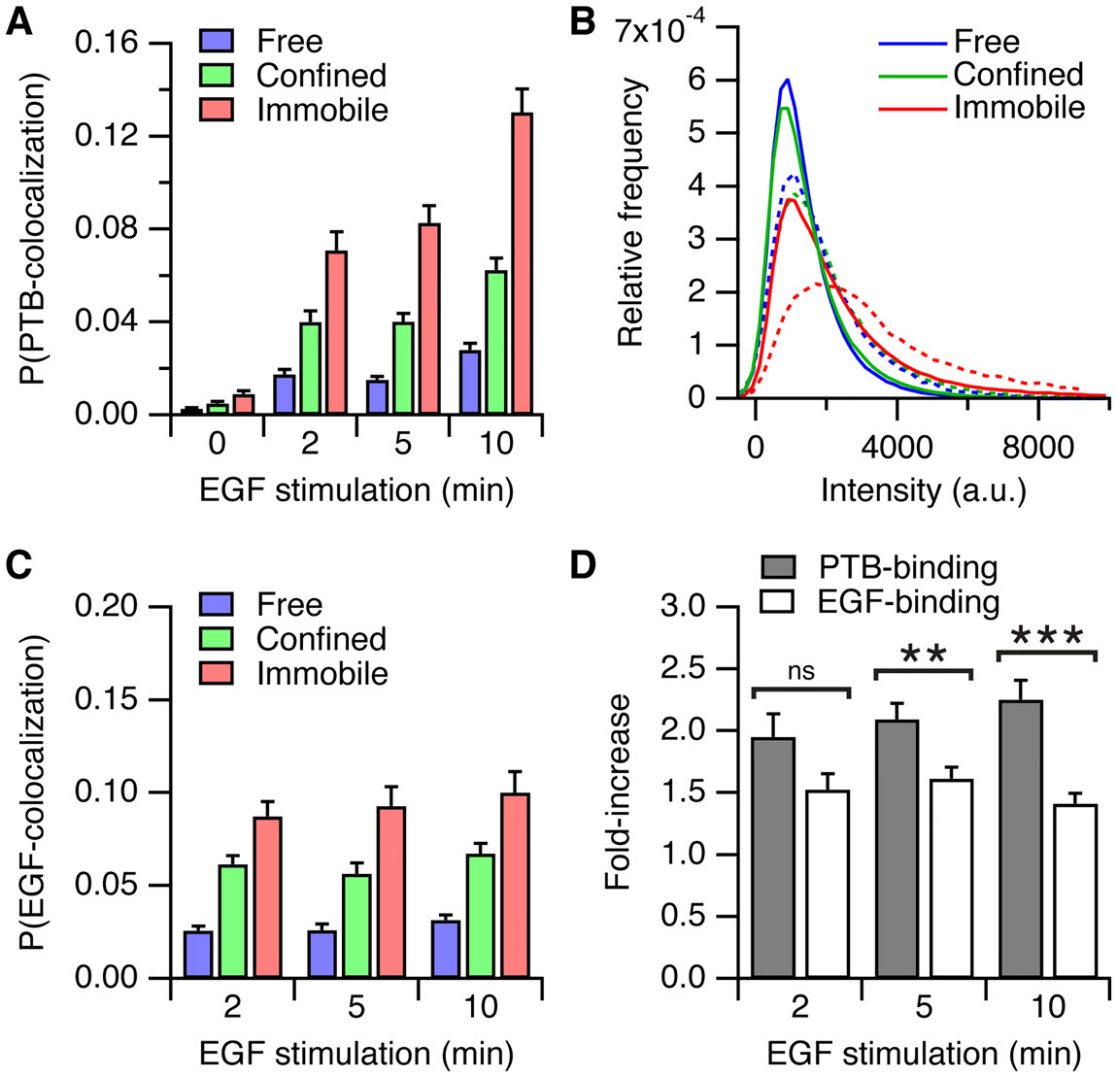
EGFR phosphorylation is amplified in the immobile state. (*A*) Probability of colocalization between Cy3-SNAP-EGFR and EGFP-PTB. (*B*) Normalized intensity histograms of the Cy3-SNAP-EGFR intensity, for all particles (*continuous lines*) and for the particles that colocalized with EGFP-PTB (*dashed lines*) after 10 minutes of EGF stimulation. (*C*) Probability of colocalization between Cy3-SNAP-EGFR and EGF-Alexa488. (*D*) Fold-increase of the colocalization probability in the immobile versus the confined state, for EGFP-PTB colocalization (see *A*), and for EGF-Alexa488 colocalization (see *C*). *A*, *B*: *n* = 43 cells; *C*, *D*: *n* = 36 cells. ns: *P* > 0.05, **P < 0.01, ***P < 0.001, *t* test. Error bars denote SEM.

We then investigated the signaling activity of EGFR by detecting the colocalization of EGFP-PTB particles with Cy3-SNAP-EGFR (Fig 5A). In this case, the colocalization probability can be interpreted as a measure of signaling activity, because it represents the likelihood that the C-terminal tyrosine residues in an EGFR particle are phosphorylated. After ligand binding, the immobile EGFR particles showed a significantly higher probability of PTB binding compared to the confined receptors, indicating that they had a higher signaling activity. Fluorescence intensity histograms of the whole population of Cy3-SNAP-EGFR and of particles that colocalized with EGFP-PTB showed that the phosphorylated receptors were brighter in the immobile state, indicating a higher degree of aggregation of the phosphorylated sub-population (Fig 5B). Bright phosphorylated particles were also observed after inhibition of protein tyrosine phosphatases with pervanadate, demonstrating that aggregation of phosphorylated EGFR was independent of ligand binding (S9 Fig).

The immobile particles have a higher signaling activity compared to free and confined states, because they represent clusters of multiple receptors (see also Fig 4). Indeed, after 10 minutes of stimulation, the PTB colocalization probability in the immobile state was twice that of the confined state (Fig 5A). However, the difference between the probabilities of EGFR aggregation in the immobile and confined states was less pronounced (Fig 4A). This suggested that immobile EGFR was phosphorylated to a higher degree than expected from its level of aggregation. To investigate this, we determined the relation between EGF binding and the phosphorylation of the receptor. We imaged Cy3-SNAP-EGFR after stimulation with EGF-Alexa488 (S4 Movie) and quantified EGF binding by estimating the probability that an EGF particle colocalized with an EGFR particle (Fig 5C). EGF mostly bound to receptors in the confined and immobile states, with the highest colocalization probability in the immobile state, due to the availability of multiple binding sites in the immobile particles. EGF binding did not change significantly after two minutes, indicating that ligand binding is not a rate-limiting step in the phosphorylation reaction. Also here, the difference in PTB binding between the immobile and confined states of EGFR was relatively large when compared to the difference in EGF binding (compare Fig 5A–5C), indicating that the phosphorylation of the receptor was not proportional to the ligand occupancy. To quantify this observation, we calculated in each cell the fold-increase of the colocalization probability of EGF or PTB in the immobile state compared to the confined state (Fig 5D). In the immobile state the colocalization probability of EGF binding was ~50% higher compared to the confined state, whereas the colocalization probability of PTB was 100% more. Immobile EGFR particles are therefore more efficiently binding effectors at their C-terminal tyrosine residues, relative to the extent of ligand binding, as compared to the confined receptors. Thus, the higher level of PTB binding in the immobile state compared to the confined state cannot be explained by a higher number of ligand-bound receptors in immobile particles, which points to an amplification of phosphorylation in receptor clusters.

### Activation of EGFR is supported by clustering in clathrin-coated pits

**Fig 6:**
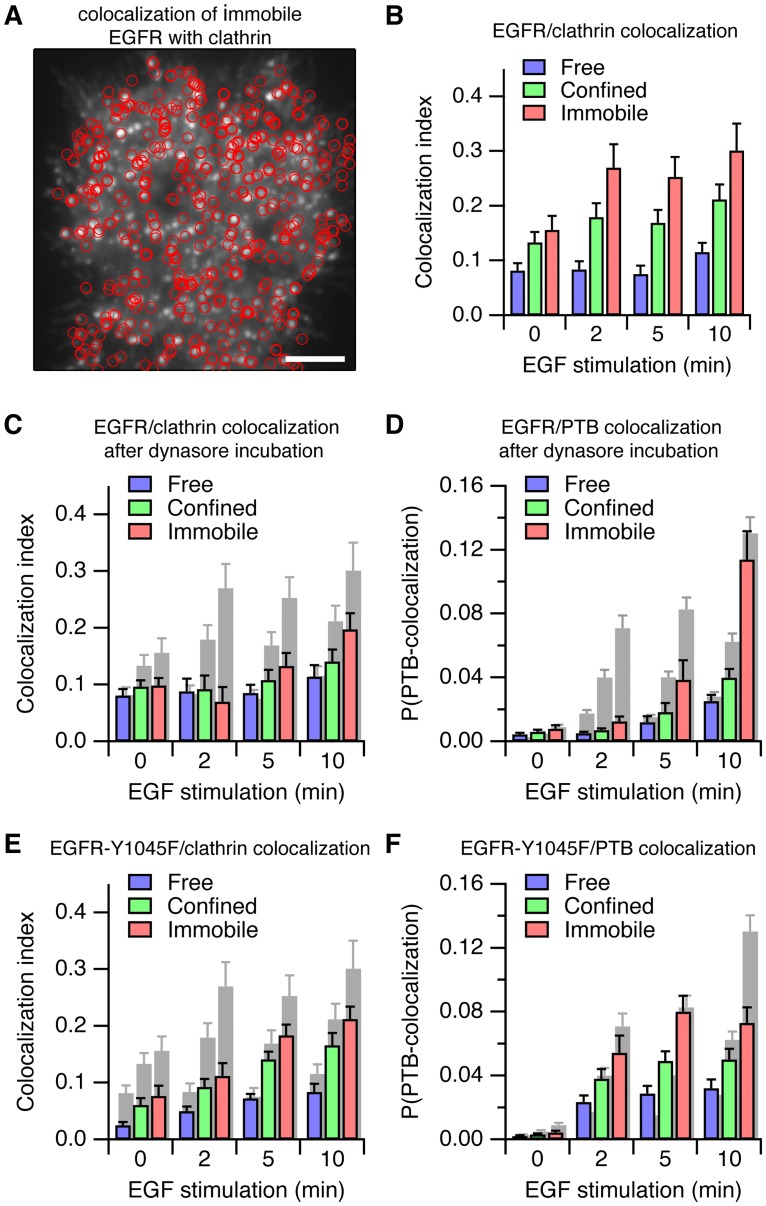
Recruitment to clathrin-coated pits is required for robust EGFR activation. (*A*) Overlay of the locations of immobile Cy3-SNAP-EGFR (red circles) and the EGFP-clathrin fluorescence (grey-scale image) in MCF-7 cells after 10 minutes of stimulation with 16 nM EGF. (*B*) Quantification of the colocalization of Cy3-SNAP-EGFR particles with EGFP-clathrin. (*C*) Colocalization between Cy3-SNAP-EGFR and EGFP-clathrin, after 30 minutes of incubation with 80 μM dynasore. (*D*) Colocalization probability between Cy3-SNAP-EGFR and EGFP-PTB, after 30 minutes of incubation with 80 μM dynasore. (*E*) Colocalization between Cy3-SNAP-EGFR-Y1045F and EGFP-clathrin. (*F*) Colocalization probability between Cy3-SNAP-EGFR-Y1045F and EGFP-PTB. *A*–*D*: *n* = 16 cells per time point, *E*, *F*: *n* = 20 cells per time point. *C*–*F*, grey bars: reproduction of the results for wild-type SNAP-EGFR in the absence of dynasore (Figs 5A and 6B). Error bars denote SEM.

Activated EGFR is recruited to clathrin-coated pits [45] where it forms clusters of up to 5 EGFR molecules [22]. Therefore we investigated if amplification of EGFR phosphorylation requires receptor clustering in clathrin-coated pits. We tracked the receptor while simultaneously imaging EGFP-tagged human clathrin light chain A (EGFP-clathrin, Fig 6A and S5 Movie). To quantify the colocalization of the EGFR particles with the EGFP-clathrin signal, we modified an existing approach for measuring the overlap of two images [41]. After EGF stimulation, immobilized EGFR particles, and to a lesser degree also confined particles, were colocalizing strongly with the EGFP-clathrin fluorescence signal in comparison to the free receptors (Fig 6B). Autonomous activation of the receptor by inhibition of protein tyrosine phosphatases with pervanadate also led to an increase in colocalization, showing that phosphorylation of the receptor was responsible for the recruitment of EGFR to clathrin-coated pits (S10 Fig). Note that not all immobile particles appeared to colocalize with EGFP-clathrin (Fig 6A), suggesting that other independent mechanisms could also operate to immobilize EGFR.

To further investigate if the amplification of the phosphorylation of EGFR occurs in the clathrin-coated pits, we interfered acutely with the development of clathrin-coated pits using dynasore, a small molecule inhibitor of dynamin [46]. Dynasore inhibits separation of clathrin-coated pits from the plasma membrane and arrests them at early growth phases [46] because their maturation is controlled by regulatory checkpoints that depend on dynamin-2 [47]. After incubation with dynasore, the colocalization of immobile SNAP-EGFR particles with EGFP-clathrin was impaired (Fig 6C, compare with Fig 6B). Since the number and size of the clathrin-coated pits were unchanged (S11 Fig), we concluded that this was the result of arresting the clathrin-coated pits in a non-functional state. We then measured the phosphorylation of EGFR particles after incubation with dynasore and found that the response was delayed (Fig 6D, compare with Fig 5A). We conclude that EGFR is not efficiently retained in clathrin-coated pits after interfering with their maturation, and as a result the amplification of the phosphorylation of EGFR is reduced, leading to a delay in the overall response to ligand.

To influence the localization of EGFR without chemically perturbing the clathrin-coated pits, we used a mutant of EGFR (Y1045F) that does not bind directly to the E3 ubiquitin-ligase c-Cbl [48]. Recruitment of EGFR to clathrin-coated pits is partially regulated via ubiquitination by c-Cbl [49]. Therefore, we expected the localization of this mutant to clathrin-coated pits to be reduced, thereby affecting the amplification of its phosphorylation. Indeed, we found that the response of this mutant to EGF stimulation, both in terms of its colocalization with EGFP-clathrin and of its phosphorylation, was reduced compared to the wild-type receptor (Fig 6E and 6F compare with Figs 5A and 6B). The mutated receptor did not reach the same level of activity as wild-type EGFR after 10 minutes of stimulation, and clustered substantially less in the immobile state (S12 Fig compare with Fig 5B).

### EGFR clusters in clathrin-coated pits are sources of signaling activity

**Fig 7:**
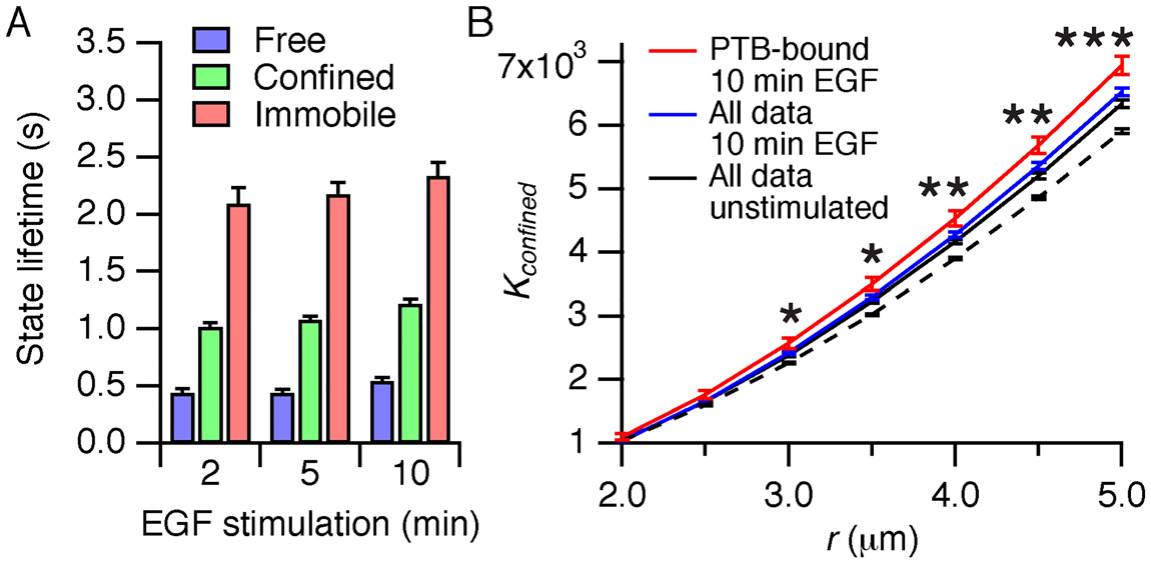
Cross-phosphorylation of EGFR establishes phosphorylation gradients. (*A*) Lifetime of the mobility states, after selecting Cy3-SNAP-EGFR particles that colocalized with EGFP-PTB (*B*) Quantification of the distribution of confined particles around immobile points (*K*_*confined*_) calculated for all particles in unstimulated and stimulated cells and for particles that colocalized with PTB after stimulation with EGF. The dashed line represents the *K*_*confined*_ function in unstimulated cells after randomization of the particle locations. *n* = 43 cells per time point. *P < 0.05, **P < 0.01, ***P < 0.001, *t* tests comparing the *K*_*confined*_ values of particles that colocalize with PTB after 10 minutes (*red line*) with the corresponding *K*_*confined*_ values of all particles before stimulation (*continuous black line*). Error bars denote SEM.

Because clathrin-coated pits are well-defined immobile structures with a lifetime of approximately one minute [50], we asked if amplification of EGFR phosphorylation could affect the signaling activity of the receptor in the surrounding plasma membrane. After ligand stimulation, the state lifetimes of Cy3-SNAP-EGFR particles that colocalized with EGFP-PTB were on a seconds time-scale (Fig 7A, compare with Fig 3B), indicating that phosphorylated particles still alternated rapidly between the different mobility states. This indicated that phosphorylated receptors in immobile aggregates were able to escape to the free and confined state. As a result of the amplification of phosphorylation in immobile EGFR particles, gradients in the signaling activity of confined and free receptors could be established [51]. In this case, the immobile EGFR particles represent sources where receptors are phosphorylated, while dephosphorylation can occur anywhere in the plasma membrane (including the clathrin-coated pits). To test for the presence of such gradients, we employed a variation of Ripley’s *K* function, a descriptive statistic that is commonly used for detecting deviations from spatial homogeneity [40]. It quantifies the average particle density in a local area around each point in a spatial pattern as a function of the area size. We adapted Ripley’s *K* function to quantify the local density of free or confined particles around immobile particles. After stimulation, the concentration of confined phosphorylated receptors was significantly increased around immobile EGFR particles, compared to the distribution of all particles in unstimulated cells (Fig 7B). For free particles a similar trend was observed, but the differences were not statistically significant (S13 Fig), either because relatively few particles are phosphorylated in the free state (Fig 5A) thereby increasing the measurement error, or because the faster diffusion of free particles leads to shallower gradients [51] that are more difficult to detect. Note that Ripley’s *K* function can be used to detect a non-random distribution, but is not suitable for inferring its properties without further knowledge. In this particular case, it demonstrates that the concentration of phosphorylated receptors is higher around the immobile particles, thereby demonstrating the existence of a gradient of phosphorylation. It does not give us further quantitative information, such as the extent of the gradient. However, the existence of these gradients shows that the observed amplification of PTB binding (Fig 5) did not just reflect an increase in effector binding efficiency, but is due to cross-phosphorylation between EGFR. The existence of phosphorylation gradients for confined receptors also shows that cross-phosphorylation in receptor clusters leads to gradients of signaling activity that localize the EGFR signal around clathrin-coated pits.

## Discussion

**Fig 8:**
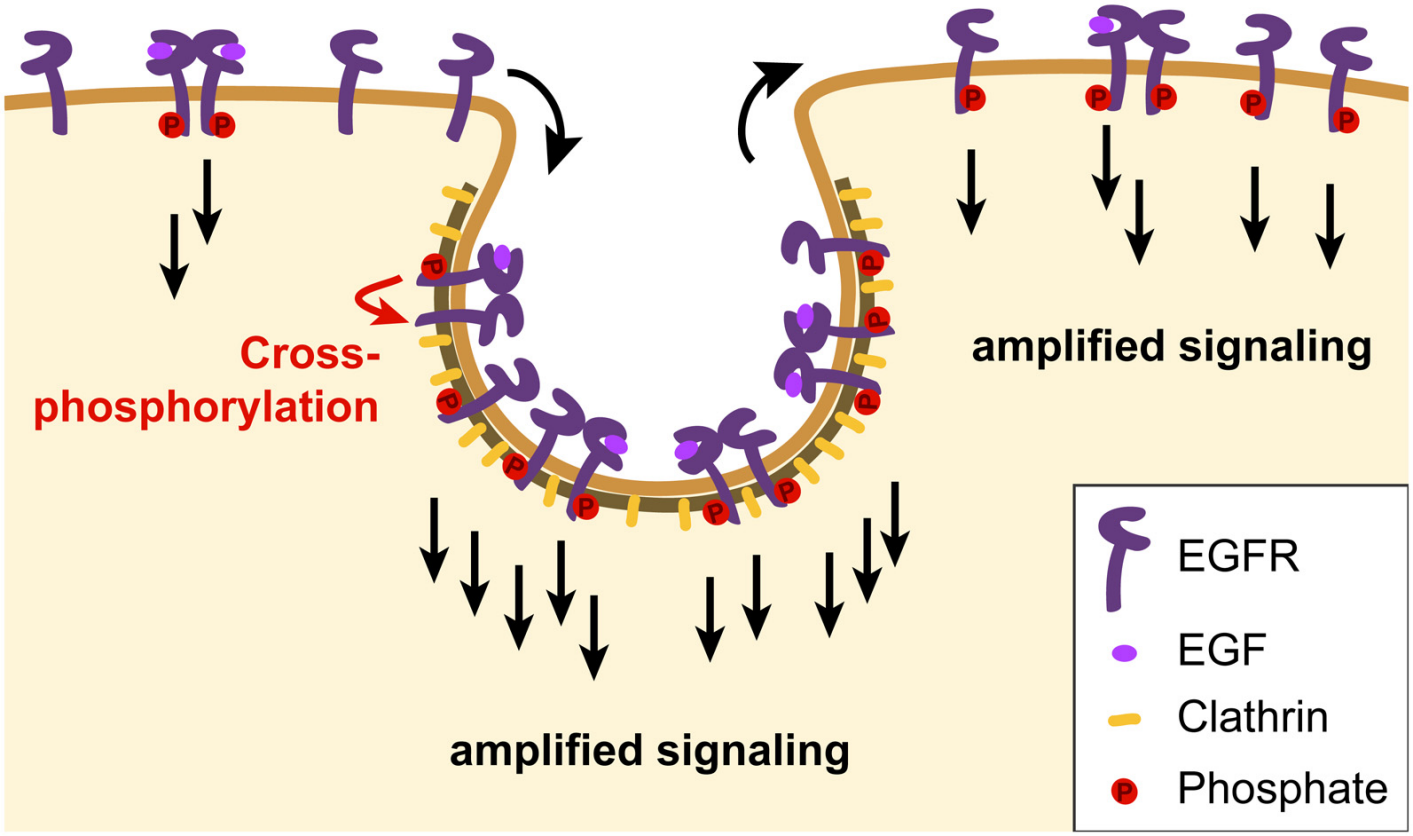
A new role for clathrin-coated pits in the early phases of EGFR signaling. Upon stimulation with ligand, epidermal growth-factor receptors are recruited to clathrin-coated pits, where their phosphorylation is amplified by clustering. Phosphorylated receptors are able to escape the clathrin-coated pits leading to an amplified EGFR signal in surrounding plasma membrane.

We employed single particle tracking to quantify the mobility, aggregation and signaling activity of EGFR at the plasma membrane, and found that its phosphorylation is amplified by receptor clustering in clathrin-coated pits. We propose that this contributes to a rapid and robust response, which establishes a new role for clathrin-coated pits in the early phases of EGFR signaling at the plasma membrane, adding to their well-established role in clathrin-mediated endocytosis, which propagates, and eventually terminates, the signal in the interior of the cell (Fig 8).

Our work introduces a novel method for quantifying the signaling activity of EGFR at a single molecule level by detecting the binding of a genetically engineered probe to phosphorylated tyrosine residues in the cytosolic tail of the receptor. In the present work we focused on the signaling activity of immobile EGFR in clathrin-coated pits, but our experimental approach is well suited to investigate other questions such as the role of the cytoskeleton in the signaling activity of EGFR. Moreover, this method is generic as it could easily be adapted for use with different receptors, by using a binding domain derived from an appropriate effector.

Our work is in agreement with earlier reports that use single molecule tracking to investigate the mobility of EGFR upon activation by ligand [15, 16]. We also find that upon stimulation with ligand the mobility of the receptor decreases markedly. In particular, we find a population of confined active receptors, lending support for the conclusions of Low-Nam et al. [16], who showed that co-confinement of the receptor by the cytoskeleton enhances dimer formation. Earlier single molecule work did not focus on the role of immobile receptors, although Chung et al. [15] reported a very slow state, which they hypothesized to be active receptors that started to interact with cytosolic proteins involved in signaling and endocytosis. Our work extends these reports by investigating the clustering and signaling activity of the receptor in the immobile state.

Clustering of EGFR has been described by several authors [17–21,23], and was reported to occur in clathrin-coated pits [22]. However, these reports did not provide a clear mechanism for cluster formation and did not clarify the functional role of these clusters. There have been no structural studies that account for EGFR clustering by direct interactions between the receptors. Phosphorylation on the regulatory tyrosine residue Tyr845 has been shown to increase the dimerization affinity of EGFR [25], but there is no evidence that this also supports higher order clustering. Our results indicate that the clustering of phosphorylated EGFR in clathrin-coated pits is predominantly dictated by effector binding. Activated EGFR binds to the clathrin adaptor AP-2 [45,52,53], providing potential binding sites in the clathrin-coated pits. The E3 ubiquitin-ligase c-Cbl is recruited to the receptor, either by binding directly at the phosphorylated tyrosine residue Tyr1045 [48], or indirectly via Grb2 which binds to the phosphorylated Tyr1068 and Tyr1086 residues [54,55]. EGFR ubiquitination is recognized by several proteins in the clathrin-coated pits, such as the AP-2 interacting proteins epsin1 and Eps15 [56,57]. Our experiments with the Y1045F mutant of EGFR, which does not bind c-Cbl directly, show that clustering of phosphorylated EGFR is caused by the aggregation of the ubiquitinated receptor in clathrin-coated pits. As a consequence, the number of molecules in an EGFR cluster depends on the number of available binding sites in a clathrin-coated pit, which can vary substantially [50]. This might explain the lack of a clear consensus on the size of EGFR clusters in the literature.

Earlier work has shown that the signaling activity of EGFR is potentiated in endosomes, as judged by a high stoichiometry of Grb2 binding to EGFR [58]. This is consistent with our findings that EGFR phosphorylation is amplified in clathrin-coated pits, which could result in an increased signaling activity of EGFR in the endocytic pathway. Our results demonstrate that EGFR signaling activity is already amplified at the initial step of clathrin-mediated endocytosis. The amplification of EGFR phosphorylation by clustering in clathrin-coated pits is likely important at low-expression levels where the encounter rate of receptors in the plasma membrane is low. Amplification of phosphorylation could also establish a positive feedback loop between EGFR aggregation and signal amplification because the recruitment of receptors to clathrin-coated pits depends on the phosphorylation of EGFR. We find that EGFR binds transiently to clathrin-coated pits, which is in agreement with a recent report that the interactions of clathrin-coated pits with other cargo can be highly transient [50]. As a result, phosphorylated receptors are able to escape to the plasma membrane. Because receptor phosphorylation is amplified in clathrin-coated pits, this leads to the formation of local gradients of signaling active receptors. This could enhance signaling from EGFR in the vicinity of clathrin-coated pits and spatially limit the response to a local stimulus. Paradoxically, these gradients could at the same time contribute to a more efficient termination of the overall EGFR signal by increasing the probability of internalizing phosphorylated receptors. Thus, the amplification of receptor phosphorylation in clathrin-coated pits establishes local gradients of signaling activity at the plasma membrane that could spatially and temporally constrain the EGFR signal.

Our data do not exclude the possibility that EGFR is also immobilized in other membrane domains apart from clathrin-coated pits. Localization to membrane rafts or confinement by the cytoskeleton could provide the same benefit of locally concentrating the receptor, and possibly enable amplification of receptor phosphorylation. The presented tools for quantifying the signaling activity of EGFR at a single molecule level should be well suited to investigate these possibilities.

Our results highlight the multi-layered nature of the intrinsic and extrinsic mechanisms that govern EGFR signal activation. Various structural features control receptor dimerization upon ligand binding, which is the key initial event that activates EGFR signaling. In addition, extrinsic mechanisms that involve intracellular factors such as tyrosine kinase phosphatases suppress autocatalytic activity, while complementary amplification mechanisms, for instance based on local phosphatase inhibition, support a stable signal upon ligand binding. Our work identifies receptor clustering in clathrin-coated pits as an additional amplification mechanism that supports activation of the EGFR signal after ligand stimulation.

## Supporting Information

S1 Fig Recruitment of EGFP-PTB to Cy3-SNAP-EGFR after stimulation with 16 nM EGF in a MCF-7 cell. Scale bar is 10 μm.
(TIF)

S2 Fig TIRF imaging of MCF-7 cells expressing SNAP-EGFR, labeled with Cy3 and stimulated with 16 nM EGF-Alexa488. (*A*) Cy3 SNAP-EGFR and EGF-Alexa488 fluorescence images for one representative example. (*B*) Pearson’s correlation coefficient between the Cy3 SNAP-EGFR and EGF-Alexa488 signals as a function of stimulation time. *n* = 8 cells. Error bars denote SEM. Scale bar is 10 μm.
(TIF)

S3 Fig Relative expression levels and densities of SNAP-EGFR in single particle tracking experiments. (*A*) The average fluorescence intensity of Cy3-SNAP-EGFR in cells selected for single particle tracking, compared to a random population of cells. The bottom and the top of the box represent the 25^th^ and 75^th^ percentiles, the bottom and top whiskers represent the 10^th^ and 90^th^ percentiles. The red line represents the median value and the cross represents the mean value. Assuming an average expression level similar to a published EGFR-EGFP construct of 5×10^5^ receptors [14], the selected cells express around 1.3×10^5^ receptors. Note that the selected cells do not display the large variation in expression levels seen in the total population. The total number of cells was 80, from which 25 were selected as suitable for tracking analysis. (*B*) Histogram of the average number of Cy3-SNAP-EGFR particles per μm^2^ in the tracking experiments.
(TIF)

S4 Fig Classified single particle tracks of Cy3-SNAP-EGFR acquired in MCF 7 cells. Each track segment is color-coded according its state (*blue*: free; *green*: confined; *red*: immobile). Black circles mark the localizations of the detected EGFP-PTB particles. The insets are magnifications of the indicated areas. (*A*) Tracks derived from an unstimulated cell (see Fig 1B). (*B*) Tracks derived from a cell that was stimulated for 10 minutes with 16 nM EGF (see Fig 1C).
(TIF)

S5 Fig MSD analysis of SNAP-EGFR diffusion. Shown is the MSD analysis of the diffusion of Cy3-SNAP-EGFR upon stimulation with 16 nM EGF, after classification of the data by mobility state. *Straight lines*: linear fits to the first 5 points of each curve. Legend: diffusion coefficients (*D*, μm^2^s^-1^) derived from these fits. *n* = 43 cells per time point. Error bars denote SEM.
(TIF)

S6 Fig MSD analysis of SNAP-EGFR diffusion for models consisting of three, four or five states. Shown is the MSD analysis of the diffusion of Cy3-SNAP-EGFR before stimulation and after 10 minutes of stimulation with 16 nM EGF, after classification of the data by mobility state. *n* = 43 cells per time point. Error bars denote SEM.
(TIF)

S7 Fig Results of the vbSPT algorithm before and after stimulation with 16 nM EGF. *Circles*: percentages of particles in the state (state occupations). *Arrows*: probabilities to switch to another state between frames (transition probabilities). *Dashed arrows* indicate transition probabilities < 0.01.
(TIF)

S8 Fig Aggregation of EGFR after incubation with 1 mM pervanadate. (*A*) Probability of Cy3-SNAP-EGFR colocalization with Alexa488-SNAP-EGFR as a function of the pervanadate incubation time. (*B*) Normalized intensity histograms of Cy3-SNAP-EGFR, for all particles (*continuous lines*) and for the particles that colocalized with Alexa488-SNAP-EGFR (*dashed lines*) after 30 minutes of pervanadate incubation. *n* = 10 cells per time point. Error bars denote SEM.
(TIF)

S9 Fig Signaling activity of EGFR after incubation with 1 mM pervanadate. (*A*) Probability of Cy3-SNAP-EGFR colocalization with EGFP-PTB as a function of the pervanadate incubation time. (*B*) Normalized intensity histograms of Cy3-SNAP-EGFR, for all particles (*continuous lines*) and for the particles that colocalized with EGFP-PTB (*dashed lines*) after 30 minutes of pervanadate incubation. *n* = 16 cells per time point. Error bars denote SEM.
(TIF)

S10 Fig Quantification of the colocalization of Cy3-SNAP-EGFR particles with the EGFP-clathrin signal after incubation with 1mM pervanadate. *n* = 16 cells per time point. Error bars denote SEM.
(TIF)

S11 Fig Distribution of EGFP-clathrin with and without dynasore treatment. (*A*) Representative images of cells expressing EGFP-clathrin without and with treatment with 80 μM dynasore for 30 minutes. (*B*) The spot density (number of fluorescent spots per μm^2^). (*C*) Average fluorescence intensity of the spots. (*D*) Average size of the spots in μm^2^. *n* = 32 cells. Error bars denote SEM.
(TIF)

S12 Fig Normalized intensity histograms of the Cy3-SNAP-EGFR-Y1045F intensity. For all particles (*continuous lines*) and for the particles that colocalized with EGFP-PTB (*dashed lines*) after 10 minutes of EGF stimulation.
(TIF)

S13 Fig Quantification of the distribution of free particles around immobile points (*K*_*free*_). Calculated for all particles in unstimulated and stimulated cells and for particles that colocalized with PTB after stimulation with EGF. The *dashed line* represents the *K*_*free*_ function in unstimulated cells after randomization of the particle locations. Error bars denote SEM.
(TIF)

S1 Movie Dual-color single molecule imaging of Cy3-SNAP-EGFR and EGFP-PTB in an unstimulated cell, and after 10 minutes stimulation with 16 nM EGF. Scale bar is 5 μm.
(AVI)

S2 Movie Overlays of the detected tracks on the acquired data of CY3-SNAP-EGFR for an unstimulated cell, and after 10 minutes of stimulation with 16 nM EGF. (AVI)

S3 Movie Dual-color single molecule imaging of Cy3-SNAP-EGFR and Alexa488-SNAP-EGFR in an unstimulated cell, and after 10 minutes stimulation with 16 nM EGF. Scale bar is 5 μm. Note the strong bleaching in the Alexa488 channel, which causes a loss of the background signal, and leads to an increase in the contrast with clustered Alexa488-SNAP-EGFR. This gives the visual impression of an increased signal in aggregates compared with the Cy3 channel, but does not indicate increased clustering.
(AVI)

S4 Movie Dual-color single molecule imaging of Cy3-SNAP-EGFR and EGF-Alexa488 after 10 minutes stimulation with 16 nM EGF-Alexa488. Scale bar is 5 μm.
(AVI)

S5 Movie Dual-color imaging of Cy3-SNAP-EGFR and EGFP-Clathrin after 10 minutes of stimulation with 16 nM EGF. Scale bar is 5 μm.
(AVI)
