# Supplementary figures and images for: Single Particle Tracking Reveals that EGFR Signaling Activity Is Amplified in Clathrin-Coated Pits
Source: PLoS One. 2015 Nov 17;10(11):e0143162. doi: 10.1371/journal.pone.0143162 (PMC4648588; doi:10.1371/journal.pone.0143162)

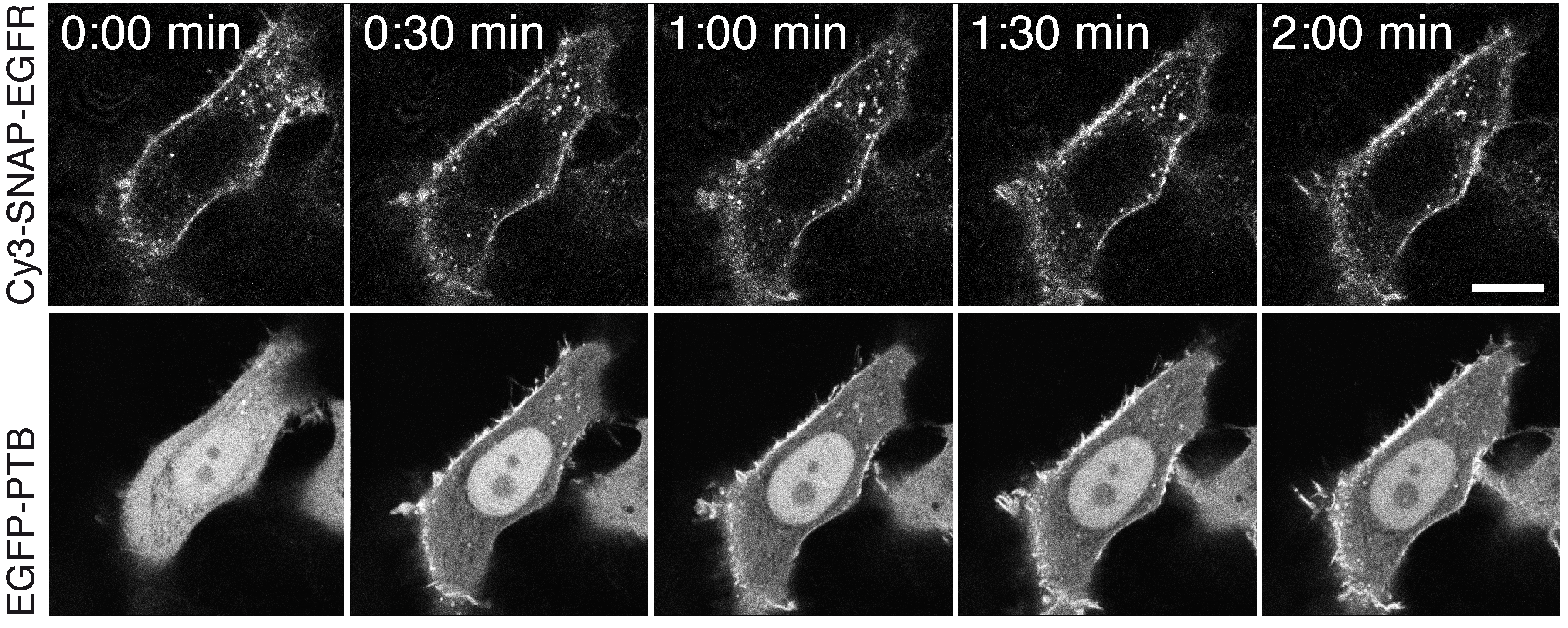

Supplement: S1 Fig — Scale bar is 10 μm. (TIF) [file pone.0143162.s001.tif]

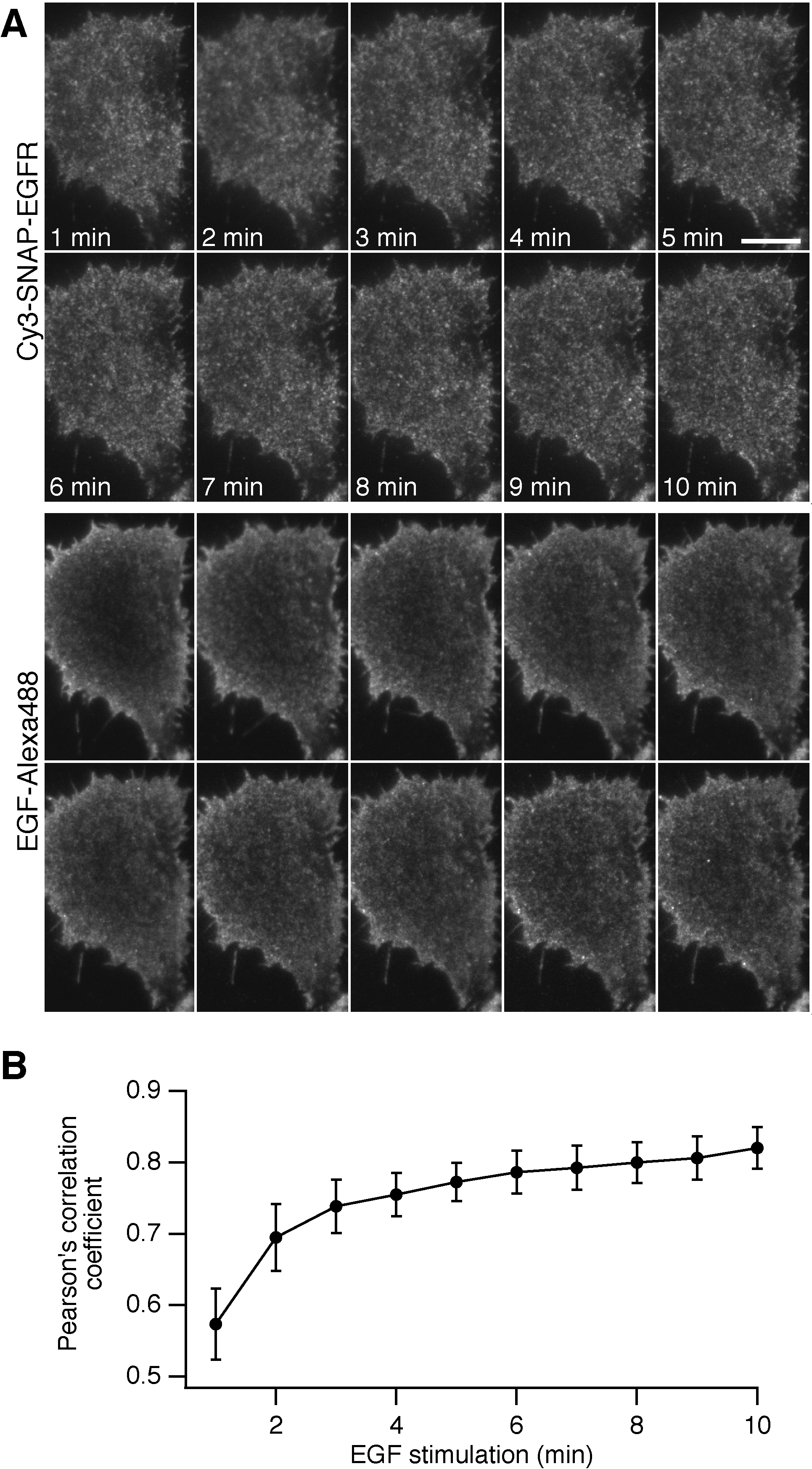

Supplement: S2 Fig — (A) Cy3 SNAP-EGFR and EGF-Alexa488 fluorescence images for one representative example. (B) Pearson’s correlation coefficient between the Cy3 SNAP-EGFR and EGF-Alexa488 signals as a function of stimulation time. n = 8 cells. Error bars denote SEM. Scale bar is 10 μm. (TIF) [file pone.0143162.s002.tif]

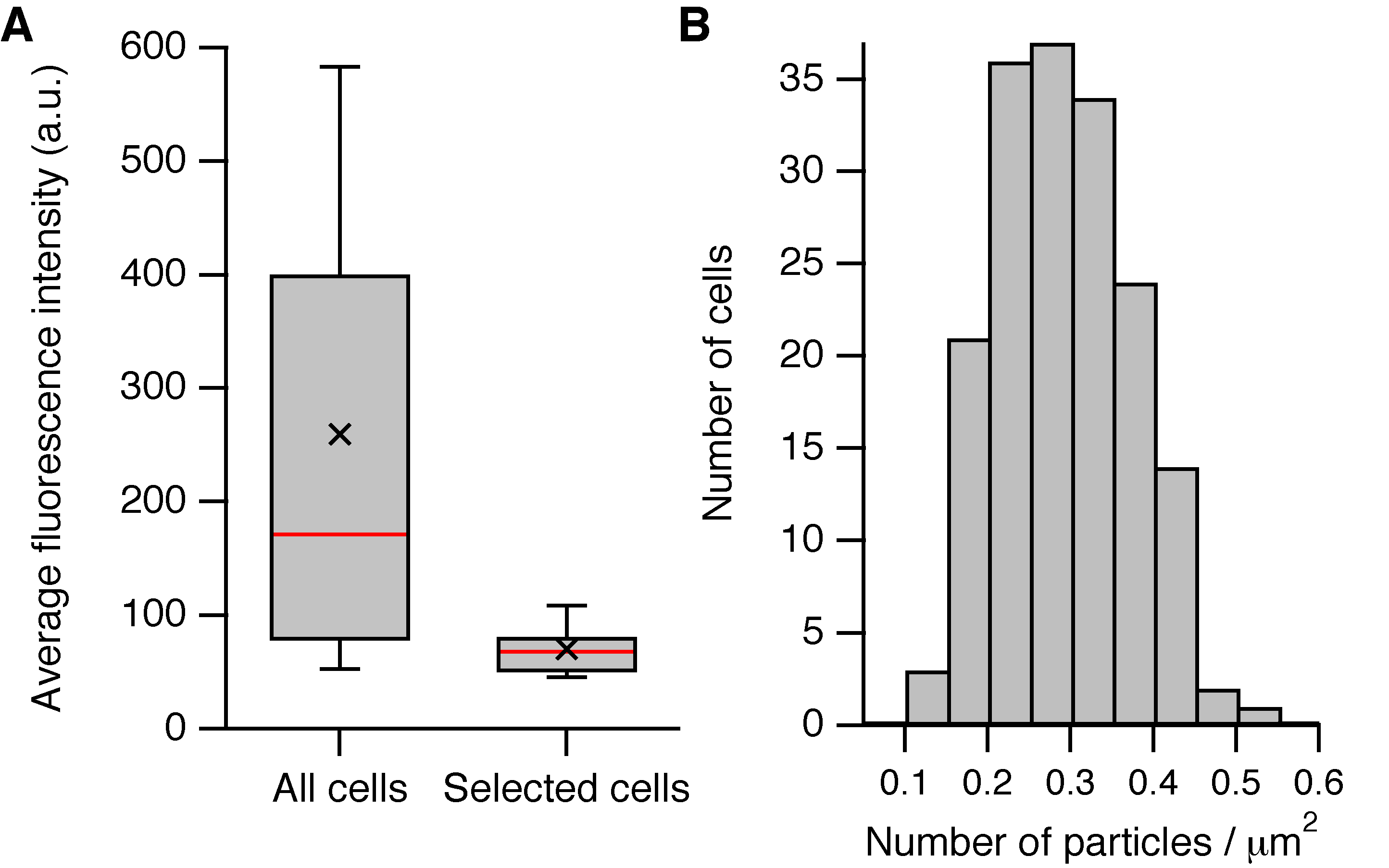

Supplement: S3 Fig — (A) The average fluorescence intensity of Cy3-SNAP-EGFR in cells selected for single particle tracking, compared to a random population of cells. The bottom and the top of the box represent the 25th and 75th percentiles, the bottom and top whiskers represent the 10th and 90th percentiles. The red line represents the median value and the cross represents the mean value. Assuming an average expression level similar to a published EGFR-EGFP construct of 5×10^5^ receptors [14], the selected cells express around 1.3×10^5^ receptors. Note that the selected cells do not display the large variation in expression levels seen in the total population. The total number of cells was 80, from which 25 were selected as suitable for tracking analysis. (B) Histogram of the average number of Cy3-SNAP-EGFR particles per μm2 in the tracking experiments. (TIF) [file pone.0143162.s003.tif]

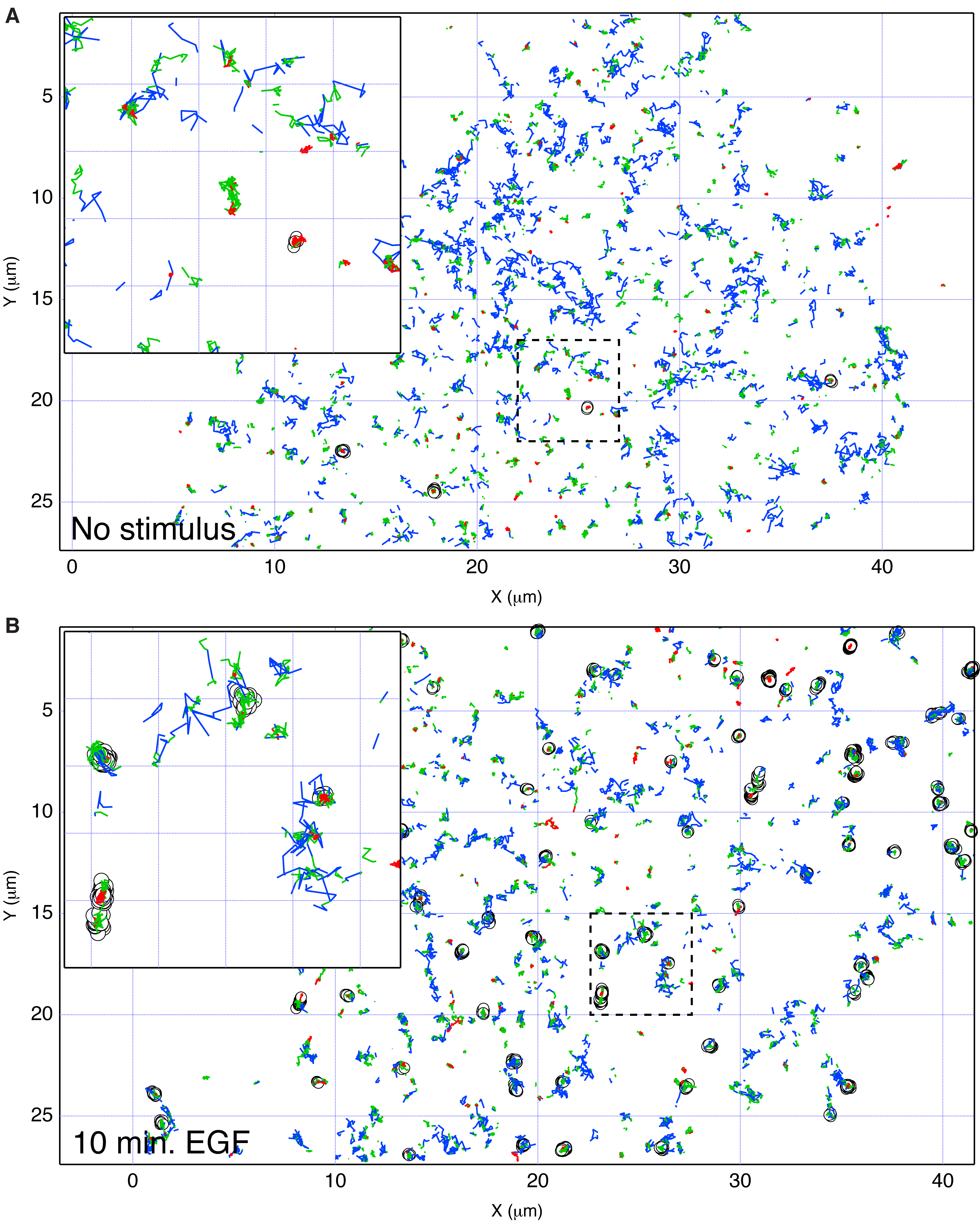

Supplement: S4 Fig — Each track segment is color-coded according its state (blue: free; green: confined; red: immobile). Black circles mark the localizations of the detected EGFP-PTB particles. The insets are magnifications of the indicated areas. (A) Tracks derived from an unstimulated cell (see Fig 1B). (B) Tracks derived from a cell that was stimulated for 10 minutes with 16 nM EGF (see Fig 1C). (TIF) [file pone.0143162.s004.tif]

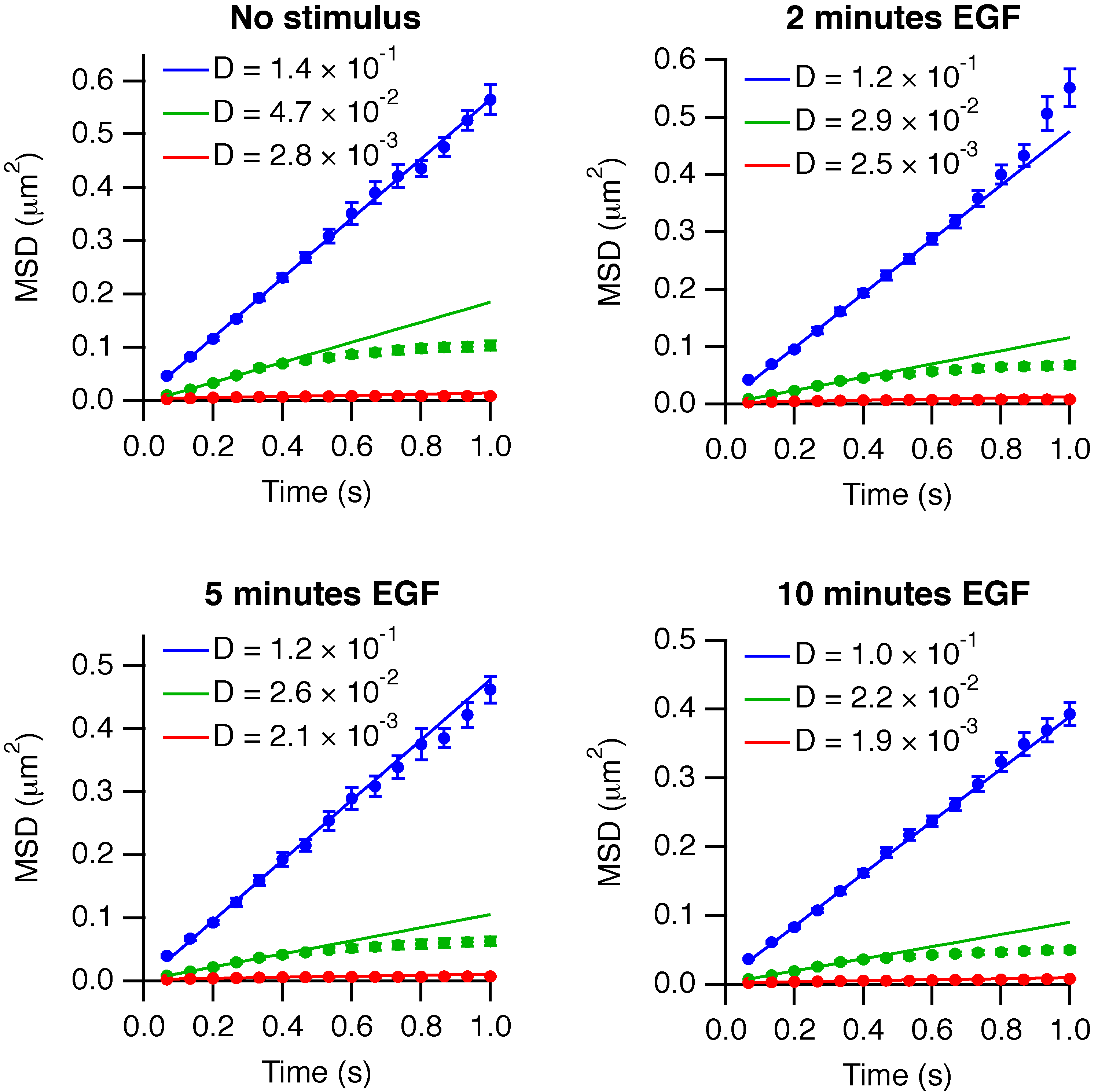

Supplement: S5 Fig — Shown is the MSD analysis of the diffusion of Cy3-SNAP-EGFR upon stimulation with 16 nM EGF, after classification of the data by mobility state. Straight lines: linear fits to the first 5 points of each curve. Legend: diffusion coefficients (D, μm2s-1) derived from these fits. n = 43 cells per time point. Error bars denote SEM. (TIF) [file pone.0143162.s005.tif]

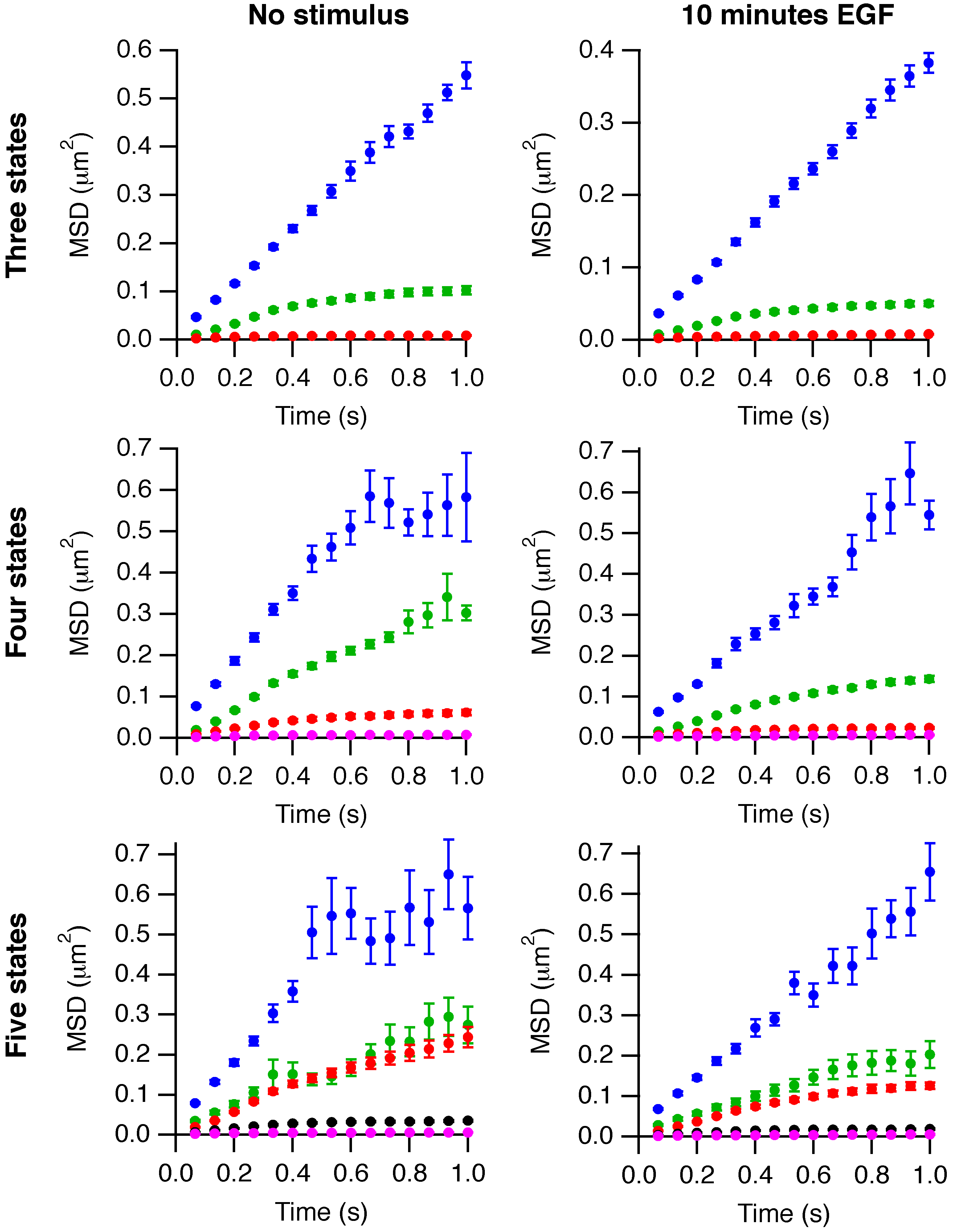

Supplement: S6 Fig — Shown is the MSD analysis of the diffusion of Cy3-SNAP-EGFR before stimulation and after 10 minutes of stimulation with 16 nM EGF, after classification of the data by mobility state. n = 43 cells per time point. Error bars denote SEM. (TIF) [file pone.0143162.s006.tif]

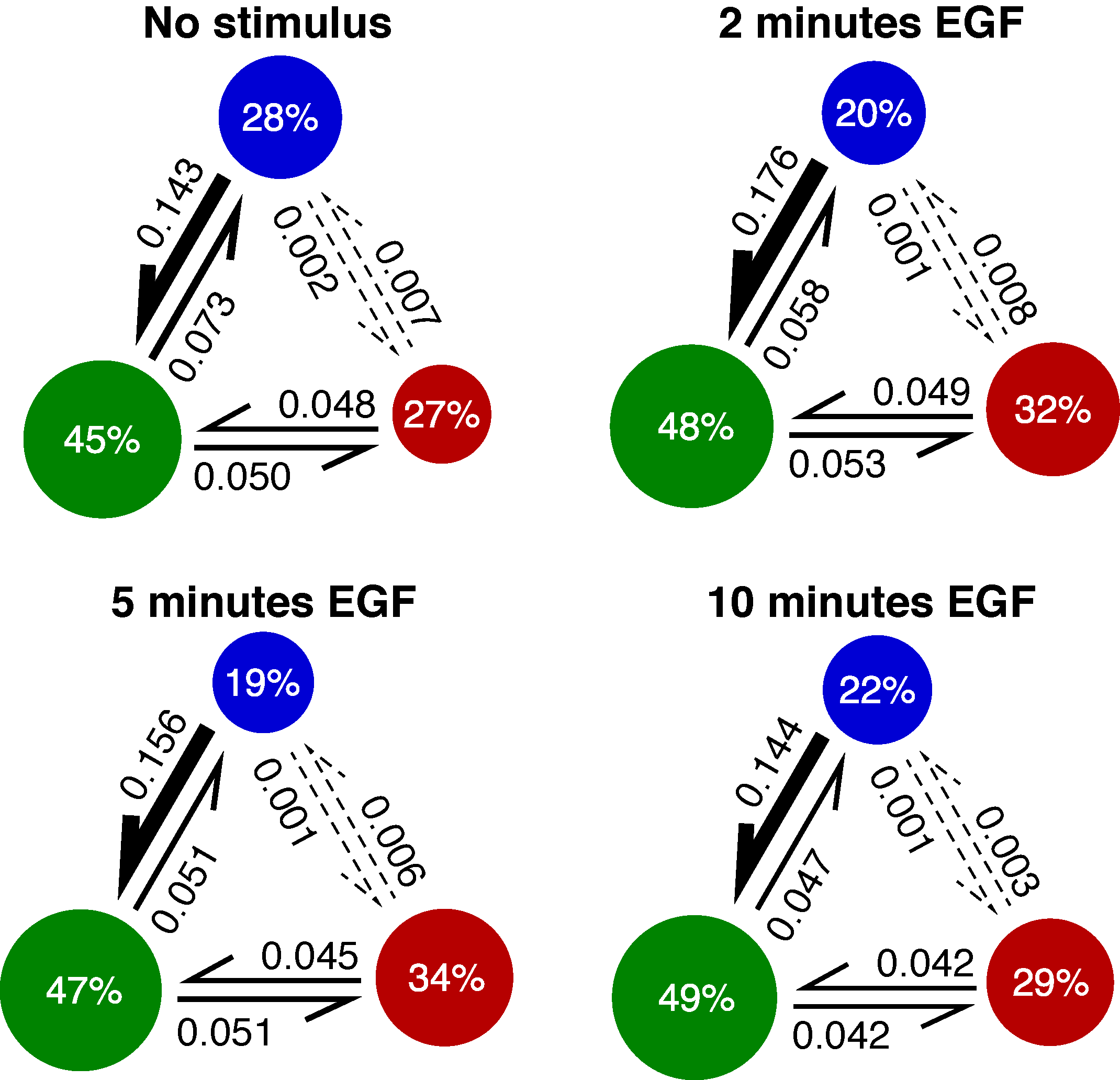

Supplement: S7 Fig — Circles: percentages of particles in the state (state occupations). Arrows: probabilities to switch to another state between frames (transition probabilities). Dashed arrows indicate transition probabilities < 0.01. (TIF) [file pone.0143162.s007.tif]

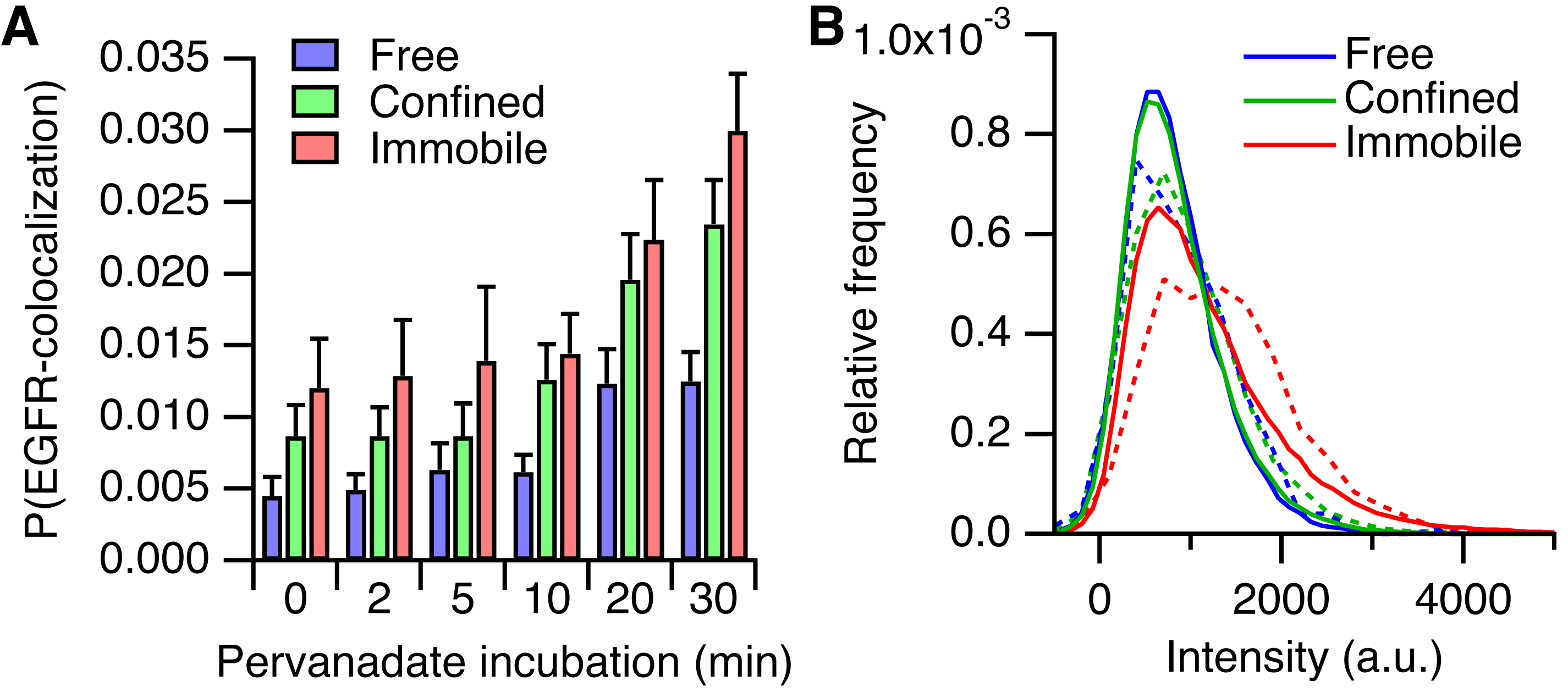

Supplement: S8 Fig — (A) Probability of Cy3-SNAP-EGFR colocalization with Alexa488-SNAP-EGFR as a function of the pervanadate incubation time. (B) Normalized intensity histograms of Cy3-SNAP-EGFR, for all particles (continuous lines) and for the particles that colocalized with Alexa488-SNAP-EGFR (dashed lines) after 30 minutes of pervanadate incubation. n = 10 cells per time point. Error bars denote SEM. (TIF) [file pone.0143162.s008.tif]

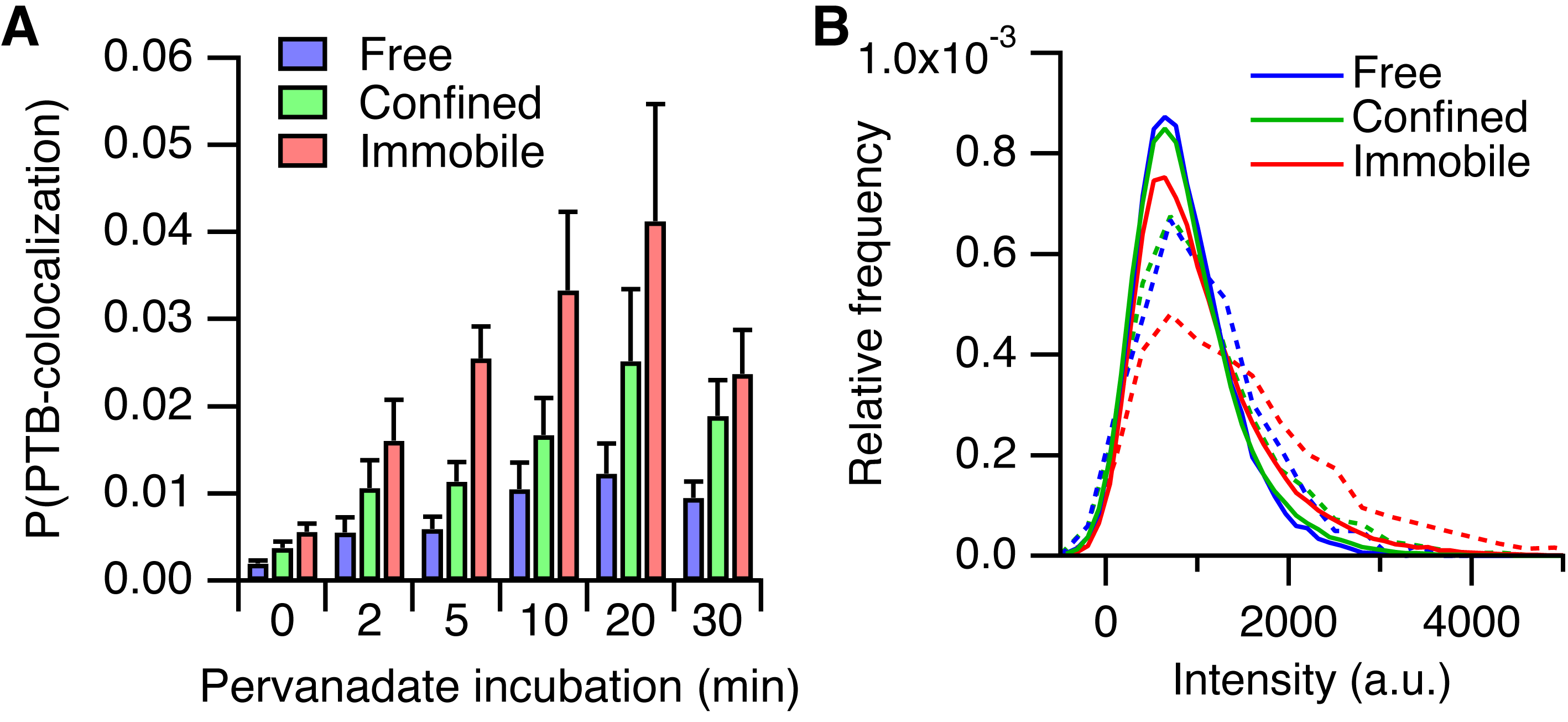

Supplement: S9 Fig — (A) Probability of Cy3-SNAP-EGFR colocalization with EGFP-PTB as a function of the pervanadate incubation time. (B) Normalized intensity histograms of Cy3-SNAP-EGFR, for all particles (continuous lines) and for the particles that colocalized with EGFP-PTB (dashed lines) after 30 minutes of pervanadate incubation. n = 16 cells per time point. Error bars denote SEM. (TIF) [file pone.0143162.s009.tif]

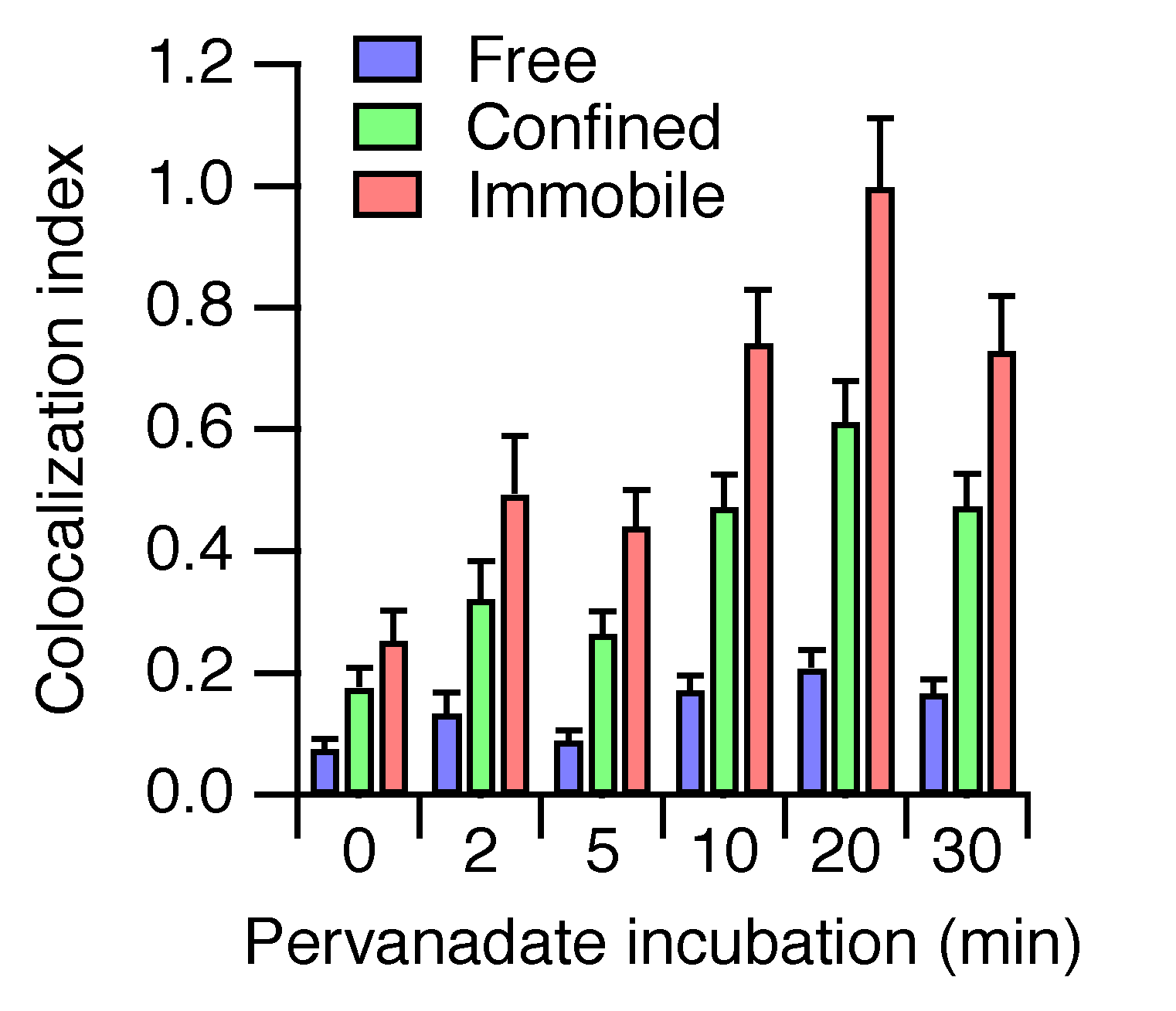

Supplement: S10 Fig — n = 16 cells per time point. Error bars denote SEM. (TIF) [file pone.0143162.s010.tif]

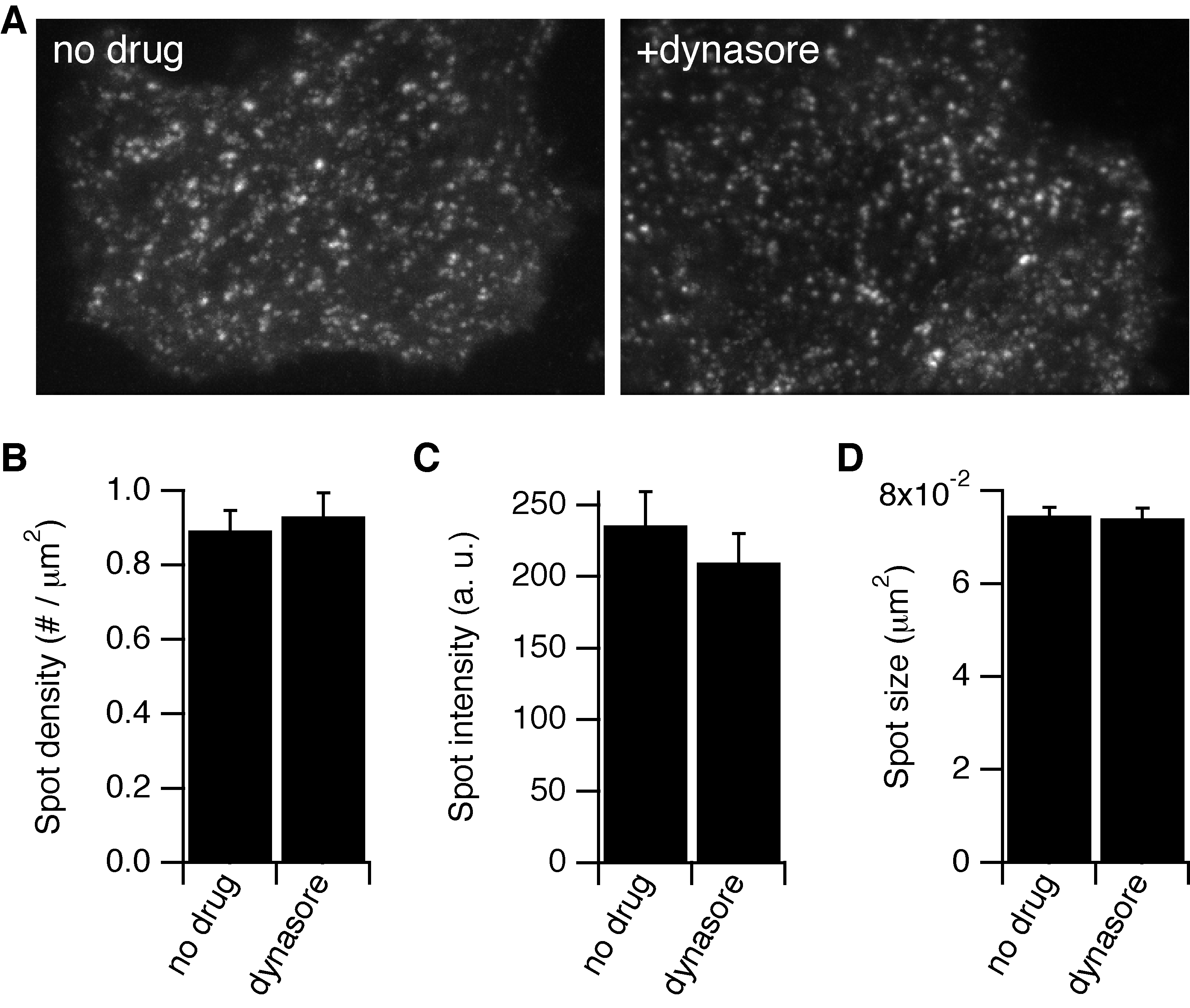

Supplement: S11 Fig — (A) Representative images of cells expressing EGFP-clathrin without and with treatment with 80 μM dynasore for 30 minutes. (B) The spot density (number of fluorescent spots per μm2). (C) Average fluorescence intensity of the spots. (D) Average size of the spots in μm2. n = 32 cells. Error bars denote SEM. (TIF) [file pone.0143162.s011.tif]

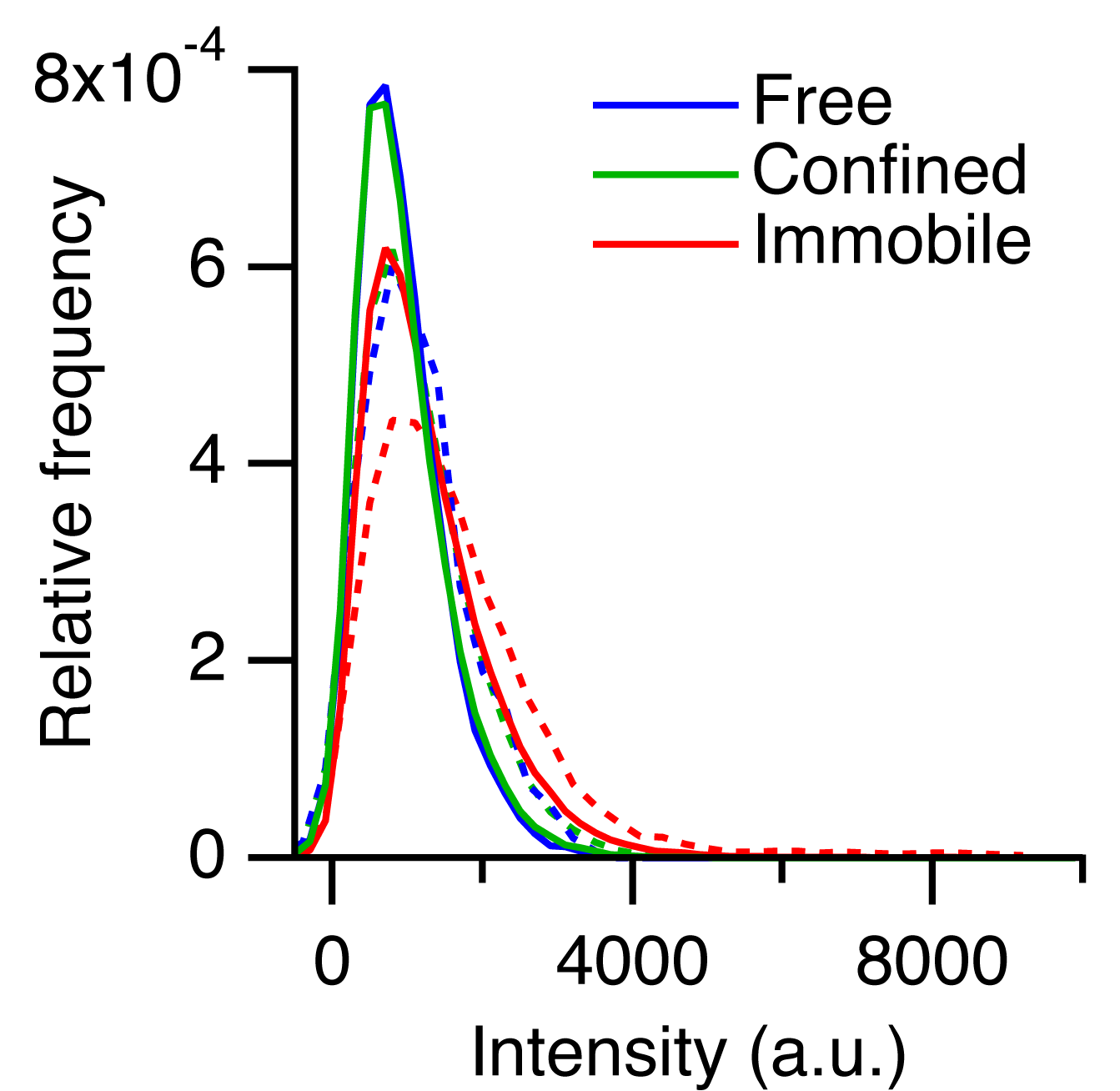

Supplement: S12 Fig — For all particles (continuous lines) and for the particles that colocalized with EGFP-PTB (dashed lines) after 10 minutes of EGF stimulation. (TIF) [file pone.0143162.s012.tif]

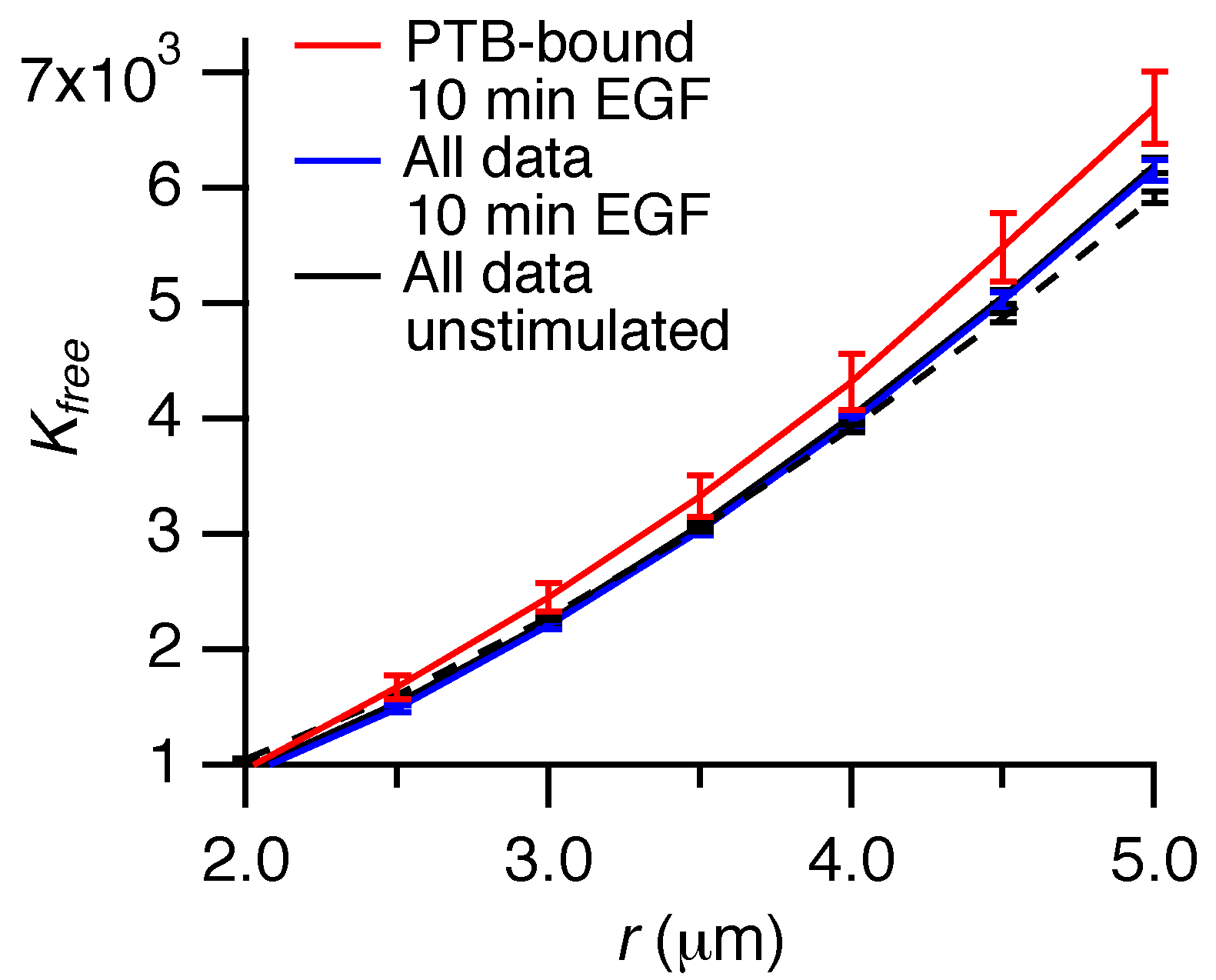

Supplement: S13 Fig — Calculated for all particles in unstimulated and stimulated cells and for particles that colocalized with PTB after stimulation with EGF. The dashed line represents the K free function in unstimulated cells after randomization of the particle locations. Error bars denote SEM. (TIF) [file pone.0143162.s013.tif]
